# Parental and personal experience drive personality formation and individual niche diversification in group-living mites

**DOI:** 10.1016/j.isci.2025.112424

**Published:** 2025-04-14

**Authors:** Thi Hanh Nguyen, Peter Schausberger

**Affiliations:** 1 Department of Behavioral and Cognitive Biology, University of Vienna, Djerassiplatz 1, 1030 Vienna, Austria; 2 Laboratory of Ecological Information, Graduate School of Agriculture, Kyoto University, Sakyo-ku, Kyoto 606-8502, Japan

**Keywords:** Biological sciences, Entomology, Zoology

## Abstract

The idea of individual niche specialization suggests that individuals should diversify in their realized niches to mitigate inter-individual conflict. We tested the hypothesis that parental and early-life diet experiences drive individual foraging specialization and animal personality formation in plant-inhabiting predatory mites *Phytoseiulus persimilis* and *Phytoseiulus macropilis*. Both species are specialized predators of herbivorous spider mites. Adult females and males, whose parents had been exposed to either prey eggs or mobile prey, and/or who themselves had experienced either eggs or mobile prey during juvenile development, were tested for their prey life stage preference, and exploration and activity patterns. Parental and/or personal experience of a given prey life stage exerted species- and sex-dependent effects on the adult predators’ mean and individual foraging phenotypes, with parental plus early-life effects being the strongest. Repeatability in activity and exploration was linked to prey life stage preference, pointing at co-variation of personality formation and individualized foraging niches.

## Highlights

• Personality of predatory mites is mediated by parental and personal experience
• Prey life stage experience modulates repeatability in activity and exploration
• The combination of parental and personal experience exerts the strongest effects
• Personalities correlate with individualized foraging niches

## Graphical abstract

**Figure:**
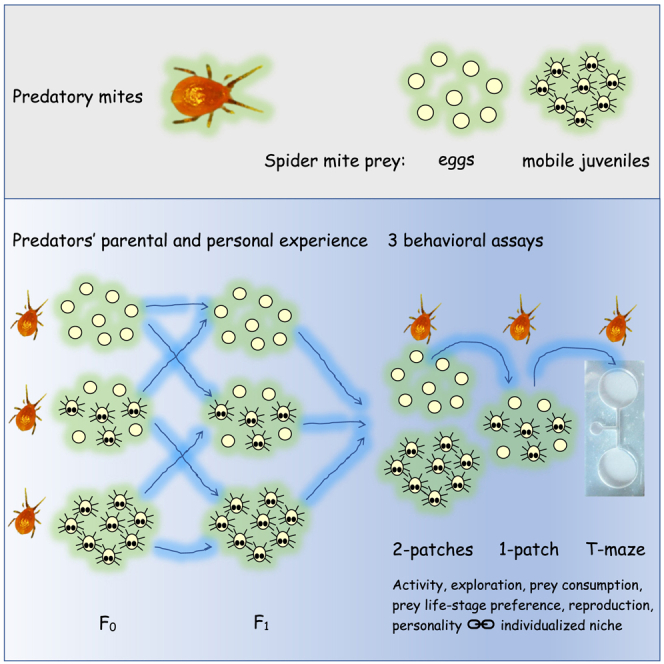

## Introduction

Animals are said to have personalities when they display consistent within-individual but variable among-individual behaviors within populations over time and across contexts.^1^^,^^2^^,^^3^^,^^4^ Five behavioral axes are commonly used to characterize animal personalities, which are activity, boldness, exploration, aggressiveness, and sociability.^3^ Personalities are ubiquitous in both vertebrates and invertebrates.^4^^,^^5^ The expression of personality can be mediated by the underlying genetic architecture and/or environmental influences.^6^ Irrespective of genetic pre-determination, environmental conditions experienced by parents (parental effects) and personal experiences during early life may play essential roles in personality formation.^7^^,^^8^^,^^9^^,^^10^ In comparison, the effects of personal experiences on animal personality are far better understood than those of parental effects. Whether and how these pathways combine and interact in personality formation remains elusive.^11^

Effects of the parental environment on the phenotype of progeny without associated changes in the DNA sequence are commonly referred to as parental effects (maternal and paternal effects) or transgenerational effects.^8^^,^^12^ Paternal effects have been largely neglected until recently, despite the fact that paternal effects are known to exist in a variety of animal taxa.^13^ Parental effects can alter the physiological development,^12^ mean behavioral trajectories and life history traits,^14^^,^^15^ and/or personality^16^^,^^17^^,^^18^^,^^19^ of offspring and can arise from one or both parents.^13^ For example, Zajitschek et al. (2017)^20^ suggested that the social conditions experienced by fathers influenced the offspring’ personalities (repeatability of activity) in zebrafish, *Danio rerio*. Horváth et al. (2019)^21^ showed that the boldness of juvenile rock lizards, *Iberolacerta cyreni*, was affected by increasing vitamin D_3_ levels in the maternal diet and corticosterone treatment early in life.

Apart from, or in addition to, the parental environment, the environment personally experienced early in life is another major pathway that can have profound effects on phenotypic development^7^^,^^22^^,^^23^^,^^24^ and, in consequence, on the expression of animal personality later in life.^3^^,^^9^^,^^10^ Several studies have reported long-lasting effects of early-life experiences on consistent behavioral variation among individuals expressed as adults. For example, snails, *Physa* *acuta*, exposed to predator cues during development were repeatable in boldness, unlike unexposed snails^25^; elevated temperature during incubation resulted in greater exploration propensity of adult males of tropical skinks, *Lampropholis similis*^26^; boldness of adult leaf beetles, *Phaedon cochleariae*, was affected by food quality during development^27^; boldness and activity emerged only in the absence of environmental stressors in mosquitofish, *Gambusia holbrooki*^28^; the landscape experienced during development affected activity and boldness of adult butterflies, *Pararge aegeria*.^29^

Prime ecological contexts in the parental generation and/or during early life affecting personality development are diet quality and/or quantity,^30^^,^^31^^,^^32^^,^^33^ predation risk,^34^^,^^35^ and social conditions.^36^^,^^37^ Two of these major ecological contexts, diet and social conditions, have also been shown to be associated with individual specialization in niche use.^38^^,^^39^^,^^40^^,^^41^^,^^42^ The theory of individual niche specialization postulates that individuals differ in the segment of the realized ecological niche covered by their population (or species) in order to mitigate intra-population competition.^39^^,^^42^^,^^43^ However, possible links between animal personality formation and individual niche specialization have rarely been addressed explicitly. Recent reviews suggest that animal personality and individual specialization in diet use and/or social conditions may be related.^44^^,^^45^ For example, the cricket *Gryllus bimaculatus*, which experienced social interactions, showed more pronounced behavioral individuality in niche use and within-individual stability than solitary-reared crickets.^46^ Han and Dingemanse (2017)^45^ observed that diet preference and personality formation in aggressiveness were driven by diet composition during juvenile development in crickets. Herath et al. (2021)^32^ discovered that the personalities of brushtail possums were linked to individual specialization in diet niches within the same landscape. The separate and interacting effects of parental and personal early-life foraging experiences on covariation of animal personality expression and individual foraging specialization are largely unexplored for any animal.

**Figure 1:**
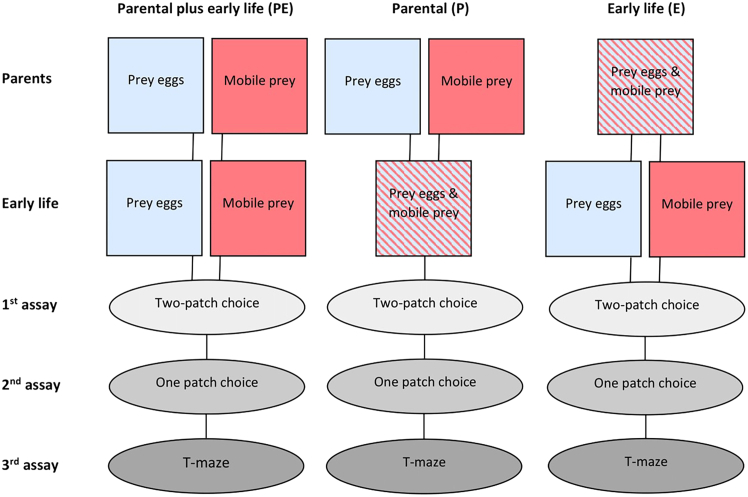
Scheme of pre-experimental treatments and behavioral assays in the parental plus early-life (PE), parental (P) and early-life (E) experiments Experimental animals were adult females and males of the predatory mites *P. persimilis* and *P. macropilis* that emerged from parents that had exclusively experienced spider mite prey as either eggs or mobile immature stages (P), or that personally experienced early in life spider mite prey as either eggs or mobile immature stages (E) or both (PE).

Here, we scrutinized the influence of parental and personal early-life experiences on the hypothesized link between individualized foraging phenotypes and personality formation in two species of group-living, plant-inhabiting predatory mites, *Phytoseiulus persimilis* Athias-Henriot and *Phytoseiulus macropilis* (Banks) (Acari, Phytoseiidae). Both species are highly specialized predators of patchily distributed herbivorous spider mites, such as two-spotted spider mites *Tetranychus urticae* Koch (Acari, Tetranychidae).^47^ Both species have a genetically pre-determined preference for the eggs over mobile life stages of their spider mite prey, although they can capture, and feed on, any life stage.^48^ In fact, a preference for spider mite eggs over mobile spider mites or vice versa is indicative of the degree of diet specialization within the family Phytoseiidae, from generalists preferring mobile prey to specialists preferring eggs.^48^^,^^49^ The specialists’ prey egg preference does not translate into immediate net benefits but delays local prey extinction.^50^ To explore the influence of parental and personal early-life experiences, alone and in combination, we varied the life stage availability of their spider mite prey. We hypothesized that the availability of different prey life stages provides information about the state of a local prey patch, which experience may lead to individual adjustment of the foraging phenotypes. The predators were exclusively offered either eggs or mobile immature stages (larvae and early protonymphs) of *T. urticae* (1) in the parental generation of the experimental animals (P), or (2) during immature development of the experimental animals themselves (personal early-life experiences; E), or (3) in both, the parental generation and during immature development of the experimental animals (PE) (Figure 1). We then assessed how different prey life stage experiences by the parents and/or during early life of the experimental animals affect their prey life stage preference and personality expression in activity and exploration as adults. We focused on activity and exploration because these are the two personality axes that are intuitively thought to be tightly linked to foraging behavior.^30^^,^^51^^,^^52^

## Results

### Population means

**Figure 2:**
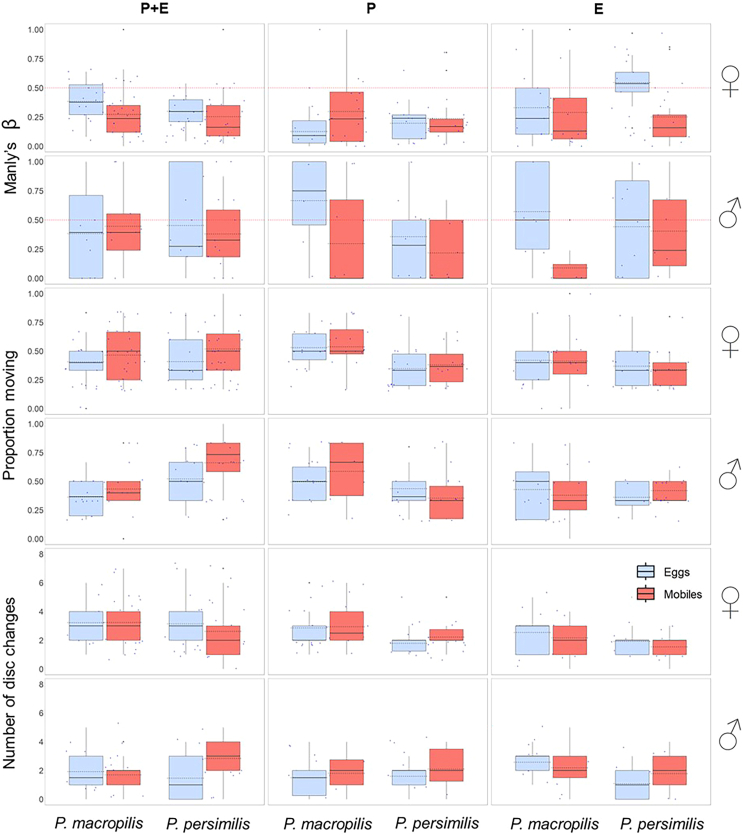
Mean prey stage preference (Manly’s β), moving activity and exploration in the two-patch choice assay of the experiments on parental plus early-life effects (PE), parental effects (P), and early-life effects (E) In the prey stage preference graphs, β > 0.5 indicates a preference for mobile spider mites while β < 0.5 indicates a preference for eggs. The broken horizontal line (β = 0.5) indicates random choice. Boxes are the 1^st^ and 3^rd^ quartiles, horizontal solid lines inside boxes are the medians, dashed lines are the means, whiskers represent the standard errors (±1), dots indicate individual data points. Statistical analysis is in Table 1.

The genetically pre-determined prey egg preference of adult *P. persimilis* and *P. macropilis* was modulated by parental and early-life experiences. Early-life experience of prey eggs weakened the egg preference (mean Manly’s β up to 0.6) in both sexes (Table 1; Figure 2), whereas parental experience of prey eggs weakened the egg preference of males (mean Manly’s β up to 0.65; Table 1; Figure 2) but strengthened the egg preference of females (mean Manly’s β < 0.25, Figure 2). In the parental plus early-life experiment, females (mean Manly’s β 0.25 to 0.4, Figure 2) had a stronger egg preference than males (mean Manly’s β 0.4 to 0.45, Figure 2). In all three experiments (parental, early-life, and parental plus early-life), the two predatory mite species had similar mean Manly’s β (Table 1; Figure 2). Mobile prey-experienced individuals showed higher mean activity than prey eggs-experienced predators in the parental plus early-life experiment (Table1; Figure 2). Mean exploration levels did not change with prey life stage experience in any of the three experiments (Figure 2). Male but not female *P. persimilis* were on average more active than *P. macropilis* in the parental plus early-life experiment, whereas *P. macropilis* was more active than *P. persimilis* in the parental experiment (Table 1; Figure 2). Males were on average less exploratory than females in the parental plus early-life and parental experiments. *P. macropilis* was more exploratory than *P. persimilis* in the early-life experiment.

**Table 1:** Generalized linear models (GLM) on the influence of prey life stage experience (eggs or mobiles; treatment), species (*P. persimilis* or *P. macropilis*), and sex on mean trait expressions in the experiments on parental plus early-life effects (PE), parental effects (P), and early-life effects (E)

Dependent variable		Independent variable	Df	*Ӽ*^*2*^	Den Df	*p*-value
Manly’s β (two-patch choice assay)	PE	Treatment	1	1.21	130	0.27
		Species	1	0.52		0.47
		Sex	1	5.14		0.02
	P	Treatment	1	1.95	90	0.16
		Species	1	1.48		0.22
		Sex	1	14.91		0.0001
		Treatment by sex	1	8.17		0.004
	E	Treatment	1	8.68	85	0.003
		Species	1	2.20		0.14
		Sex	1	0.005		0.94
Proportion moving (two-patch choice assay)	PE	Treatment	1	6.21	131	0.01
		Species	1	0.36		0.55
		Sex	1	0.49		0.48
		Species by sex	1	4.60		0.03
	P	Treatment	1	0.12	94	0.73
		Species	1	17.31		<0.0001
		Sex	1	0.24		0.62
	E	Treatment	1	0.05	86	0.83
		Species	1	0.96		0.33
		Sex	1	0.04		0.85
Number of disc changes (two-patch choice assay)	PE	Treatment	1	0.22	131	0.64
		Species	1	0.003		0.96
		Sex	1	16.77		<0.0001
	P	Treatment	1	1.14	94	0.29
		Species	1	2.22		0.14
		Sex	1	5.50		0.02
	E	Treatment	1	0.14	86	0.71
		Species	1	6.92		0.009
		Sex	1	0.42		0.52
Prey consumption (total)	PE	Treatment	1	1.14	126	0.29
		Species	1	20.79		<0.0001
		Sex	1	225.92		<0.0001
		Species by sex	1	9.50		0.002
	P	Treatment	1	0.00	90	0.99
		Species	1	38.24		<0.0001
		Sex	1	139.91		<0.0001
		Species by sex	1	13.09		0.0003
	E	Treatment	1	1.96	84	0.16
		Species	1	43.10		<0.0001
		Sex	1	120.87		<0.0001
		Species by sex	1	16.98		<0.0001
Egg production (total)	PE	Treatment	1	2.71	79	0.10
		Species	1	0.74		0.39
	P	Treatment	1	0.36	54	0.55
		Species	1	10.73		0.001
	E	Treatment	1	0.20	48	0.65
		Species	1	0.03		0.87

**Figure 3:**
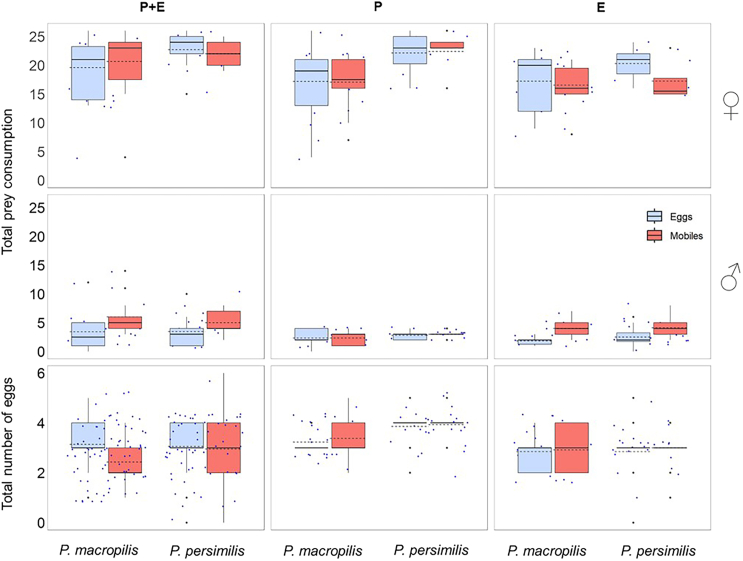
Total prey consumption (eggs and mobiles) and total number of eggs laid per female over the course of the experiments on parental plus early-life effects (PE), parental effects (P), and early-life effects (E) Experimental animals were females and males of the predatory mites *P. persimilis* and *P. macropilis* that emerged from parents (P) that had exclusively experienced spider mite prey as either eggs or mobile immature stages, or that personally experienced early in life spider mite prey as either eggs or mobile immature stages (E) or both (PE). Boxes are the 1^st^ and 3^rd^ quartiles, horizontal solid lines inside boxes are the medians, dashed lines are the means, whiskers represent the standard errors (±1), dots indicate individual data points. Statistical analysis is in Table 1.

In no experiment did the prey life stage experience affect the mean number of prey items consumed (Table 1; Figure 3). Mean prey consumption differed between sexes, predatory mite species, and their interaction in all three experiments (Figure 3). *P. persimilis* females consumed more prey items than *P. macropilis* females whereas males of the two species had similar consumption rates. Across the three behavioral assays of each experiment, *P. persimilis* females laid more eggs than *P. macropilis* females, yet this was only significant in the parental experiment (Figure 3). Prey life stage experience had no impact on egg production in any of the experiments (Table 1; Figure 3).

### Personality expression (ICCs)

**Figure 4:**
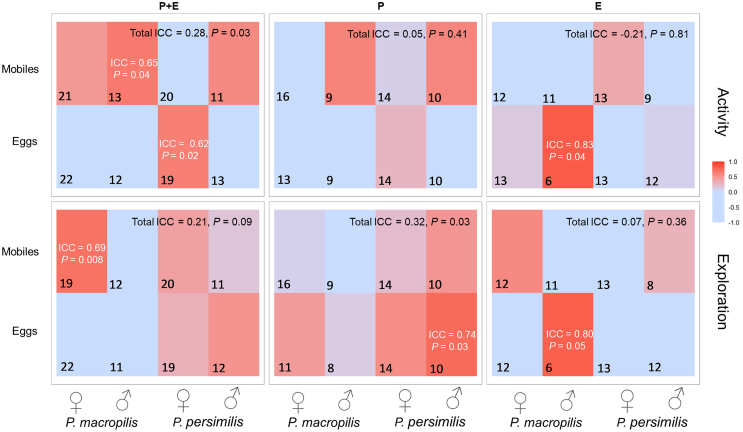
Heatmap of intraclass correlation coefficients (ICCs; repeatability) in activity and exploration in the experiments on parental plus early-life effects (PE), parental effects (P), and early-life effects (E) Experimental animals were females and males of the predatory mites *P. persimilis* and *P. macropilis* that emerged from parents (P) that had exclusively experienced spider mite prey as either eggs or mobile immature stages, or that personally experienced early in life spider mite prey as either eggs or mobile immature stages (E) or both (PE). Color and shading indicate ICCs from light blue (−1) to red (+1); sections without ICCs are non-significant (*p* > 0.1); numbers in the lower left corner of each section are the number of replicates per subgroup.

Activity of *P. persimilis* and *P. macropilis* was moderately repeatable across sub-groups when the predators had parental plus personal early-life experiences of a given prey life stage but not with either parental or early-life experience alone (Figure 4). Scrutiny of the ICCs (repeatabilities) of the sub-groups, defined by prey treatment, sex, and species, revealed that in the personal plus early-life experiment, personality expression (repeatability) in activity was most pronounced in *P. macropilis* males experienced with mobile prey and *P. persimilis* females experienced with prey eggs. In the parental experiment, *P. macropilis* males experienced with prey eggs scored the highest ICC (Figure 4). Intermediate group level analysis did not reveal any further significant ICCs in activity.

Exploration propensity of *P. persimilis* and *P. macropilis* was weakly repeatable across sub-groups in the parental plus early-life and parental experiments but not in the early-life experiment (Figure 4). Male *P. persimilis* with parental experience of prey eggs and male *P. macropilis* with early-life experience of eggs showed the highest ICC (repeatability) values in exploration. Also *P. macropilis* females that had parental plus early-life experience of mobile prey were highly repeatable in exploration (Figure 4). Intermediate group level analysis revealed that in the parental plus early-life experiment, females (species and prey stage treatments pooled: *N* = 80, *ICC* = 0.357, *p* = 0.02) and individuals with mobile prey experience (species and sexes pooled: *N* = 62, *ICC* = 0.396, *p* = 0.02) were moderately repeatable in exploration propensity.

### Within-group composition of personality types

**Figure 5:**
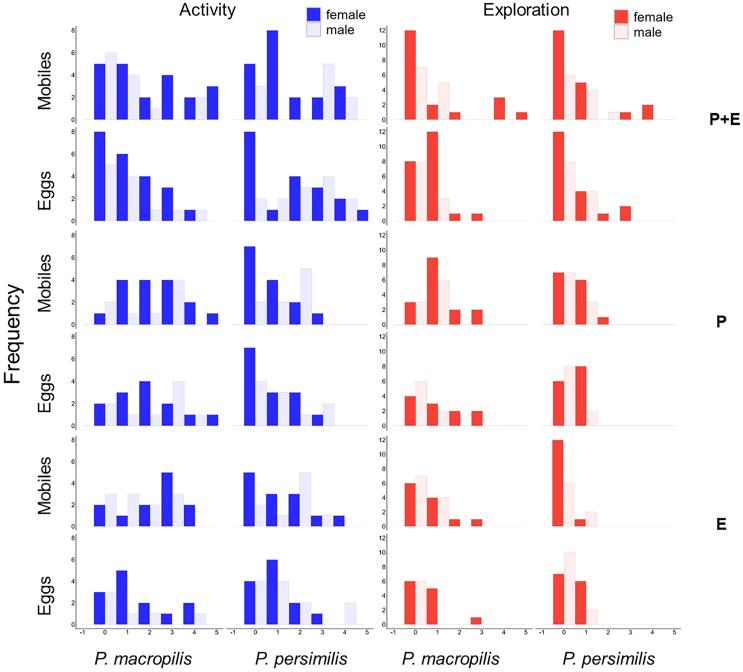
Frequency distribution of personality types in activity (blue) and exploration (red) in the experiments on parental plus early-life effects (PE), parental effects (P), and early-life effects (E) Experimental animals were females (dark bars) and males (light bars) of the predatory mites *P. persimilis* and *P. macropilis* that emerged from parents (P) that had exclusively experienced spider mite prey as either eggs or mobile immature stages, or that personally experienced early in life spider mite prey as either eggs or mobile immature stages (E) or both (PE). Higher scores indicate more active and more exploratory personality types (Table S1 for categorization of personality types); marginal scores represent the behaviorally consistent individuals. Statistical analysis is in the subsection “within-group composition of personality types” in the results section.

Within-group personality composition in activity and exploration did not vary with prey life stage experience in any of the three experiments (GLM, activity: *Ӽ*^*2*^ < 1.81, *p* > 0.18; exploration: *Ӽ*^*2*^ < 2.29, *p* > 0.13; Figure 5). Personality composition in activity varied significantly between predatory mite species in the parental experiment (*Ӽ*^*2*^ = 18.49, *p* < 0.001; Figure 5), and marginally significantly in the parental plus early-life experiment (*Ӽ*^*2*^ = 3.47, *p* = 0.06; Figure 5), with consistently more active types in *P. persimilis*. In neither experiment did the personality composition in activity differ between males and females (*Ӽ*^*2*^ < 1.28, *p* > 0.26; Figure 5). Personality composition in exploration varied between species and sexes in the parental experiment (species: *Ӽ*^*2*^ = 6.99, *p* = 0.008; sex: *Ӽ*^*2*^ = 9.21, *p* = 0.002; Figure 5) and the early-life experiment (species: *Ӽ*^*2*^ = 5.14, *p* = 0.02; sex: *Ӽ*^*2*^ = 4.71, *p* = 0.03; Figure 5): In both experiments, the distribution of personality types in exploration was biased toward more exploratory types in *P. macropilis* and females. In the parental plus early-life experiment, females had more exploratory types than males (GLM; *Ӽ*^*2*^ = 9.03, *p* = 0.003; Figure 5).

### Personality types and short-term fitness

**Figure 6:**
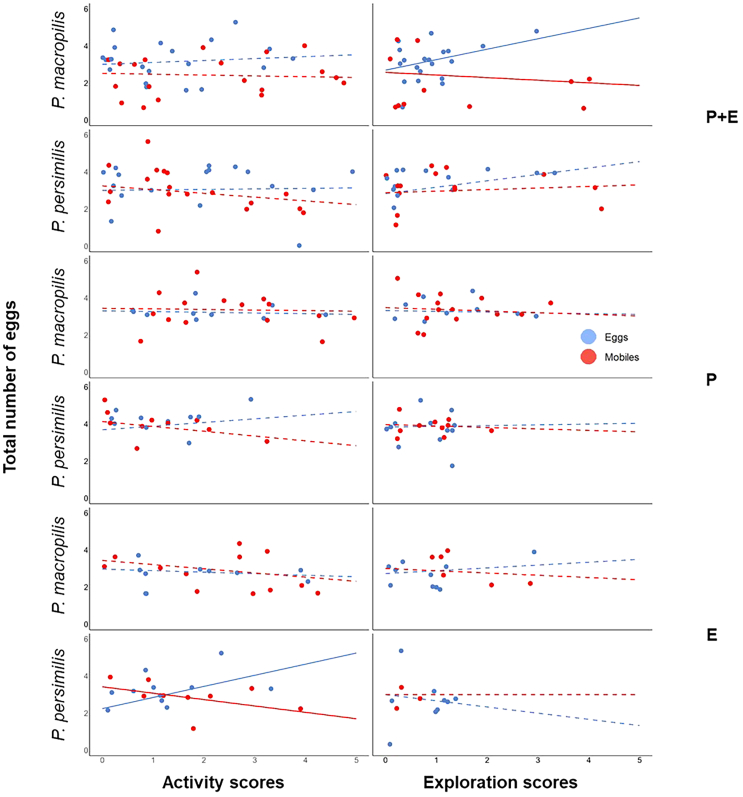
Linear regressions of the total number of eggs on the personality scores in activity and exploration in the experiments on parental plus early-life effects (PE), parental effects (P), and early-life effects (E) Experimental animals were females of the predatory mites *P. persimilis* and *P. macropilis* that emerged from parents (P) that had exclusively experienced spider mite prey as either eggs or mobile immature stages, or that personally experienced early in life spider mite prey as either eggs or mobile immature stages (E) or both (PE). Higher scores indicate more active and more exploratory personality types (Table S1 for categorization of personality types); marginal scores represent the behaviorally consistent individuals, dots indicate the individual data points; solid lines indicate significant regressions (*p* ≤ 0.05), while dashed lines indicate non-significant regressions (*p* > 0.05).

Pooled across sub-groups, personality types in activity and exploration were unrelated to egg production in the parental plus early-life experiment (linear regression; activity: *N* = 82, *r* = −0.09, *p* = 0.44; exploration: *N* = 80, *r* = 0.04, *p* = 0.73; Figure 6) and the early-life experiment (activity: *N* = 51, *r* = −0.13, *p* = 0.37; exploration: *N* = 50, *r* = −0.05, *p* = 0.73). In the parental experiment, the activity scores were marginally significantly negatively related to egg production (activity: *N* = 57, *r* = −0.24, *p* = 0.07). In contrast, there was no significant relationship between the exploration scores and egg production (*N* = 55, *r* = −0.2, *p* = 0.15; Figure 6). Sub-group-specific analysis revealed that prey eggs-experienced *P. macropilis* females with more exploratory personality types produced more eggs than less exploratory females in the parental plus early-life experiment (*N* = 22, *r* = 0.43, *p* = 0.05). In contrast, mobile prey-experienced *P. persimilis* females with more active personalities laid fewer eggs in the early-life experiment (*N* = 13, *r* = −0.55, *p* = 0.05; Figure 6). Intermediate group level analysis revealed positive correlations between exploration scores and egg production in prey eggs–experienced predators (species pooled; linear regression; *N* = 41, *r* = 0.34, *p* = 0.02) in the parental plus early-life experiment. Negative correlations between the activity scores and egg production were observed in mobile prey-experienced predators (species pooled) in the parental (*N* = 30, *r* = 0.33, *p* = 0.07) and early-life experiment (*N* = 25, *r* = 0.42, *p* = 0.03) experiments.

### Personality types and prey stage preference (Manly’s β)

**Figure 7:**
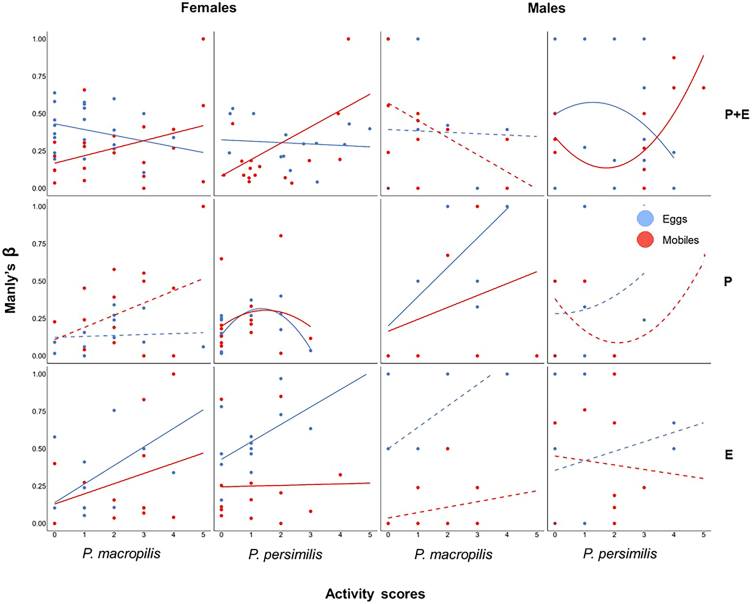
Linear and quadratic regressions (shown are the best fitting models) of prey preference (Manly’s β) on the personality scores in activity in the experiments on parental plus early-life effects (PE), parental effects (P), and early-life effects (E) Experimental animals were females (dark bars) and males (light bars) of the predatory mites *P. persimilis* and *P. macropilis* that emerged from parents (P) that had exclusively experienced spider mite prey as either eggs or mobile immature stages, or that personally experienced early in life spider mite prey as either eggs or mobile immature stages (E) or both (PE). Manly’s β > 0.5 indicates a preference for mobile spider mites while β < 0.5 indicates a preference for eggs. Higher personality scores indicate more active and more exploratory personality types (Table S1 for categorization of personality types); marginal scores represent the behaviorally consistent individuals, dots indicate the individual data points; solid lines indicate significant regressions (*p* ≤ 0.05), while dashed lines indicate non-significant regressions (*p* > 0.05).

The links between the personality scores in activity and exploration and Manly’s β varied with prey life stage experience, species and sex (Figure 7). Pooled across sub-groups, activity scores were predictive of Manly’s β in the parental experiment (linear regression; *N* = 93, *r* = 0.29, *p* = 0.004) and marginally significant in the early-life experiment (*N* = 88, *r* = 0.18, *p* = 0.08) but not in the parental plus early-life experiment (quadratic regression; *N* = 130, *r*^*2*^ = 0.02, *p* = 0.22). Sub-group-specific analysis revealed numerous significant linear or quadratic relations between the activity scores and Manly’s β (Figure 7). In the parental experiment, more active prey eggs-experienced *P. macropilis* males had a marginally significantly stronger preference for mobile prey (linear regression; *N* = 8, *r* = 0.66, *p* = 0.08). Prey eggs-experienced *P. persimilis* females showed a concave relation between Manly’s β and personality types in activity (*N* = 14, *r*^*2*^ = 0.46, *p* = 0.01; Figure 7). In the early-life experiment, more active prey eggs-experienced *P. macropilis and P. persimilis* females had a stronger preference for mobile prey (linear regression; *P. macropilis*: *N* = 13, *r* = 0.56, *p* = 0.04; *P. persimilis*: *N* = 13, *r* = 0.52, *p* = 0.07). In the parental plus early-life experiment, more active mobile prey-experienced *P. persimilis* and *P. macropilis* females had a (marginally) significantly stronger preference for mobile prey (linear regression; *P. persimilis*: *N* = 20, *r* = 0.51, *p* = 0.01; *P. macropilis*: *N* = 19, *r* = 0.37, *p* = 0.09; Figure 7), whereas mobile prey-experienced *P. persimilis* males showed a marginally significantly convex relation between Manly’s β and personality types in activity (*N* = 11, *r*^*2*^ = 0.45, *p* = 0.09; Figure 7). Intermediate group level analysis revealed positive linear correlations between activity scores and Manly’s β in *P. macropilis* (*N* = 42, *r* = 0.30, *p* = 0.04) and prey eggs-experienced (*N* = 43, *r* = 0.34, *p* = 0.02) predators in the early-life experiment, and in prey eggs- (*N* = 45, *r* = 0.10, *p* = 0.03) and mobile prey (*N* = 48, *r* = 0.28, *p* = 0.05) -experienced predators and males (*N* = 36, *r* = 0.36, *p* = 0.03) in the parental experiment. Quadratic regressions of Manly’s β on the activity scores revealed a concave relation in *P. macropilis* (prey treatments and sexes pooled; *N* = 45, *r*^*2*^ = 0.17, *p* = 0.01) in the parental experiment, and convex relations in females (species and prey treatments pooled; *N* = 82, *r*^*2*^ = 0.07, *p* = 0.04) and mobile prey-experienced individuals (species and sexes pooled; *N* = 65, *r*^*2*^ = 0.12, *p* = 0.01) in the parental plus early-life experiment.

**Figure 8:**
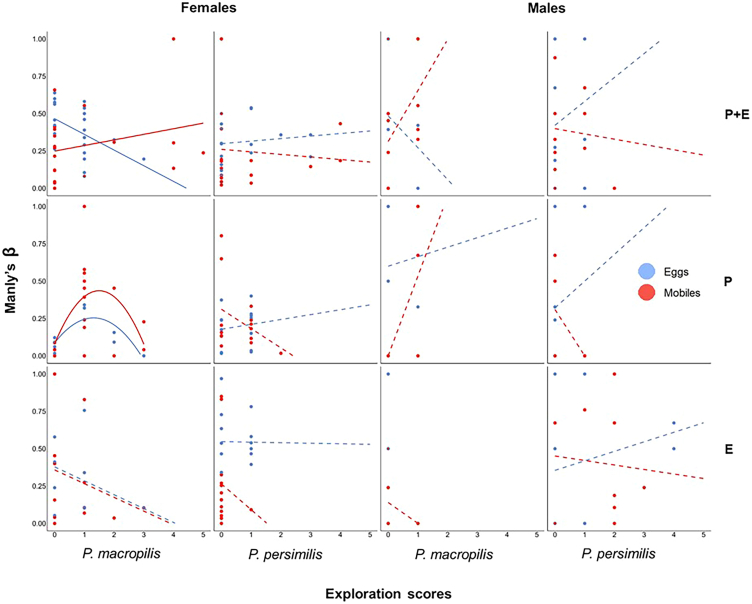
Linear and quadratic regressions (shown are the best fitting models) of prey preference (Manly’s β) on the personality scores in exploration in the experiments on parental plus early-life effects (PE), parental effects (P), and early-life effects (E) Experimental animals were females (dark bars) and males (light bars) of the predatory mites *P. persimilis* and *P. macropilis* that emerged from parents (P) that had exclusively experienced spider mite prey as either eggs or mobile immature stages, or that personally experienced early in life spider mite prey as either eggs or mobile immature stages (E) or both (PE). Manly’s β > 0.5 indicates a preference for mobile spider mites while β < 0.5 indicates a preference for eggs. Higher personality scores indicate more active and more exploratory personality types (Table S1 for categorization of personality types); marginal scores represent the behaviorally consistent individuals, dots indicate the individual data points; solid lines indicate significant regressions (*p* ≤ 0.05), while dashed lines indicate non-significant regressions (*p* > 0.05).

Pooled across sub-groups, in no experiment was the within-group composition of personality types in exploration predictive of Manly’s β (*N* = 86, 93 and 125, *p* > 0.01). In the parental plus early-life experiment, prey eggs-experienced *P. macropilis* females showed a negative correlation between Manly’s β and exploration scores (linear regression; *N* = 22, r = −0.48, *p* = 0.02, Figure 8). In the parental experiment, prey eggs- and mobile prey-experienced *P. macropilis* females showed a concave relation between Manly’s β and personality types in exploration (quadratic regression; *N* = 11, *r*^*2*^ = 0.73, *p* = 0.003 and *N* = 16, *r*^*2*^ = 0.29, *p* = 0.04; Figure 8). Intermediate group level analysis showed concave relations between the exploration scores and Manly’s β in *P. macropilis* (sexes and prey treatments pooled; *N* = 42, *r*^*2*^ = 0.14, *p* = 0.03) and females (species and prey treatments pooled; *N* = 55, *r*^*2*^ = 0.10, *p* = 0.05) in the parental experiment.

## Discussion

Our experiments demonstrate that the plant-inhabiting predatory mite species *P. persimilis* and *P. macropilis* have personalities in activity and exploration. Personality expression varied with species and sex and was contingent upon prey life stage experience. The parental and/or early-life experience of different life stages of spider mite prey modulated personality expression and generated individualized foraging phenotypes in terms of the strength of prey stage preference. The separate effects of parental and early-life effects were not simply additive but interacted in complex ways. Judged by the number of traits and sub-groups affected, the influence of prey life stage experience on personality formation and within-group personality composition linked to prey life stage preference was strongest in the parental plus early-life experiment, followed by the parental experiment. Thus, overall, parental effects were stronger modulators of personalities and their link to individualized foraging niches than early-life effects. Species and sex were stronger determinants than prey life stage experience across all mean trait expressions and experiments. Across sub-groups, determined by species, sex and prey life stage experience, parental plus early-life experience produced personalities in activity and exploration. Parental experience, but not early-life experience, produced personalities in exploration. The within-group composition of personality types in activity and exploration did not vary between prey life stage experiences across the three experiments but differed between species in both personality traits, activity and exploration, with *P. persimilis* having more active types than *P. macropilis* in the parental plus early-life and parental experiments. Females had more exploratory types than males in the parental and early-life experiments. In some sub-groups, more exploratory types had higher fitness, while more active types had lower fitness. In many subgroups, especially in the parental plus early-life and parental experiments, personality types in activity and exploration correlated with prey life stage preference, which highlights the link between personality expression and individualized niche use.

### Parental and early-life effects on mean trait expressions

Our findings corroborate that predatory mites specialized on spider mites have a genetically pre-determined preference for feeding on the egg stage of their prey.^49^^,^^53^ In *P. persimilis* and *P. macropilis*, this preference is not due to immediate nutritional benefits, because eggs and early mobile life stages of spider mites are similarly favorable for the predators in terms of life history performance.^48^^,^^49^^,^^53^^,^^54^ Modeling predator-prey dynamics suggests that a preference for prey eggs delays local prey extinction and allows more predator descendants in a local patch consisting of mixed prey life stages, as compared to no preference or preference for mobile life stages.^50^ Exposure to specific prey life stages in the parental generation or early in life subtly altered the mean prey life stage preference of the predators. In both predatory mite species, early-life experience of prey eggs weakened the mean egg preference, while parental experience of prey eggs weakened the mean egg preference of male but not female offspring. A similar trend was found in the parental plus early-life experiment. Our results confirm previous findings that not only early-life diet experience but also parental diet experience can influence the foraging behavior of offspring, even in the absence of marked differences in nutritional value but presence of marked differences in administration such as in prey item shape and movement.^55^ Several studies showed that personal diet experience alters the mean foraging behavior of predatory mites through learning.^56^^,^^57^^,^^58^^,^^59^^,^^60^^,^^61^ Parental diets changing the offspring' mean foraging behavior by prenatal learning is well documented for mammals^62^^,^^63^ but has also been observed in invertebrates such as cuttlefish^64^ and predatory mites.^55^^,^^65^ Intriguingly, in our experiments there were presumably only subtle differences, if any, in nutrient composition and flavor of the parental diet, providing little opportunity for embryonic learning, yet parental feeding on different prey life stages still induced remarkable changes in offspring' foraging preferences. Possible proximate explanations include epigenetic changes induced by parental feeding on stationary (egg) versus moving (larvae and nymphs) prey, and/or extremely fine-tuned embryonic perception of differences in yolk chemical profiles resulting from maternal feeding on spider mite eggs and mobiles, and subsequent memory and feedback loops causing changes in offspring’ foraging phenotypes as adults. Most previous studies on transgenerational diet effects have typically used stressors such as nutritional imbalance or deficiency or greatly differing diets that cause metabolic or hormonal changes passed on to offspring^12^ but none of this was the case in our experiments. Both prey conditions tested (eggs and mobile immature life stages) are nutritionally similarly favorable for the predatory mites.

Mean moving activity and exploration propensities were largely unaffected by parental and personal prey experiences in isolation, but when combined in the parental plus early-life experiment, mean activity was higher in mobile prey- than prey eggs-experienced predators. Females were more exploratory than males and consumed about three times more prey items than males. Females have much higher prey needs than males for they are twice as large and produce eggs. In order to leave prey for their offspring, females are more prone to explore nearby food sources.^66^ *P. persimilis* females fed more than *P. macropilis* females in all three experiments, which was likely due to the slightly larger body size of the former.^67^ However, we did not observe any prey life stage experience-associated differences in total prey consumption and mean egg production, a proxy for fitness. A proximate explanation is that eggs and larvae have similar nutrition benefits for the predators if available in surplus.^48^^,^^54^ Furuichi et al. (2005)^68^ observed that the reproduction rate of the predatory mite *N. womersleyi*, which is less specialized on spider mite prey, was higher when females fed on eggs than on adult females of *T. urticae*. This was explained by higher handling costs of predatory mite females when using adult spider mite females as prey. Linked to the degree of adaptation to exploit spider mites, phytoseiid predators differ in the ability to crack the chorion and the integument of moving prey.^47^ In our experiments, we supplied the predators with easy-to-capture immature spider mites, which are much easier to grasp than adult spider mites. Even if assuming that searching and handling times of individual prey items by *P. persimilis* and *P. macropilis* slightly differ between prey eggs and larvae/early protonymphs, at similar nutritional content, surplus availability, as in our experimental design, allows the predators to compensate for any such differences. Ultimately, variations in prey life stage availability lead to fine-tuned behavioral adjustment yet no reproductive consequence when females are foraging alone. At the group level, this allows for the long-term co-existence of different foraging phenotypes, driven by parental and personal foraging experiences.

### Parental and early-life effects on personality expression and within-group personality composition

The predators’ personalities in activity and exploration differed based on the prey life stage experienced by their parents and/or by themselves early in life. In many sub-groups, significant ICCs or altered within-group personality composition or co-variation with diet preferences were mediated by parental and/or early-life prey life stage experience, indicating a considerable influence of environmental stimuli associated with different prey life stages (eggs or mobile juveniles) within the same prey species. Moreover, the same stimuli sometimes differed in their effect, depending on whether they were perceived in the parental generation or personally early in life or in both phases. Parental plus personal experience of a given prey life stage shaped the personalities in activity, which was not the case for either parental or personal early life experience alone. This finding underscores that it is often only the combination of transgenerational effects and the personal experiences of offspring that leads to adaptive behavioral changes.^8^^,^^11^^,^^55^ We are not aware of any other study that compared the separate and combined effects of parental and early-life diet experiences on the formation of personality and its expression later in life. Furthermore, previous studies have mainly focused on the effects of differences in the quality or availability of food on mean trait expression and/or animal personality rather than on similarly nutritious but differently administered diets. Tremmel and Müller (2013)^27^ showed that experience with high-quality food during development increased the repeatability in activity in mustard leaf beetles. In contrast, Careau et al. (2014)^69^ observed in zebra finches that dietary restriction early in life caused greater consistency in activity. Supplementing the maternal diet with vitamin D_3_ influenced the offspring’ personality in activity in rock lizards.^21^ Royauté and Dochtermann (2017)^70^ did not observe any effects of diet quality during development on mean activity and repeatability of activity in adult crickets, while Han and Dingemanse (2017)^45^ observed that male crickets fed a high-protein diet developed more active personalities.

The predatory mites were repeatable in exploration in the parental plus early-life and the parental experiment but not in the early-life experiment. Similar to personality in activity, parental experience was the critical driver of the offspring’ personalities in exploration and had in any case a stronger influence than personal early-life experience. In the same vein, Schuett et al. (2013)^17^ observed in cross-fostering experiments that zebra finch offspring personality in exploration was mediated by the foster parents’ exploratory type but not by the parents’ genetic makeup. Yip et al. (2021)^19^ discovered paternal effects in colonial spiders, *Cyrtophora citricola*: food-restricted fathers produced offspring with more exploratory personalities than did well-fed fathers, whereas personal diet experiences did not influence personality in exploration. Krause and Naguib (2014)^71^ found stronger effects of the parental than personal experience of poor versus good nutritional conditions on exploration by zebra finches but did not assess repeatability. In their review, Langenhof and Komdeur (2018)^10^ list a few studies documenting the effects of early-life diet conditions on exploration behavior. For example, Krause et al. (2009)^72^ and Noguera et al. (2015)^73^ observed that zebra finches receiving nutritionally poor diets during early-life became on average more exploratory than individuals fed with enriched diets. However, these studies did not assess personality development in exploration. Kelleher et al. (2019)^74^ found that carotenoid-deficient diets during early life increased among- and within-individual variances but had no effect on mean and repeatable exploration of frogs, *Pseudophryne corroboree*.

### Personality expression linked to fitness

Animal personality formation and expression are highly fitness-relevant with selection acting on the underlying genetic architecture determining their expression, which includes the reaction norms and switch points if, when and how to change behavior in response to environmental stimuli.^3^^,^^75^^,^^76^ In our experiments, personalities in activity and exploration did not generally correlate with reproduction pooled across sub-groups. However, links between personality expression and fitness surfaced in some sub-groups. *P. macropilis* females experienced with prey eggs by parents combined with personal early-life experience were more reproductive when they were consistently more exploratory. In contrast, *P. persimilis* females experienced with mobile prey early in life were less reproductive when they were consistently more active. As they are able to escape, mobile prey can take a predator more time and energy to capture and handle than prey eggs. When predators are highly active, the energy expended for increased movement may come at the cost of reduced reproduction. For example, Harris et al. (2020)^77^ observed that heightened activity during winter was associated with poorer breeding performance in shy but not bold birds. The opposite was observed in red squirrels, where females showed no correlation between repeatability in activity and progeny production.^78^ Our experiments also revealed a positive correlation between personality scores in exploration and egg production in prey eggs-experienced *P. macropilis* females in the parental plus early-life experiment. Early access to new food resources by more exploratory personalities should promote reproductive success, though the early meta-study by Smith and Blumstein (2008)^76^ did not yet discover a general trend. McCowan et al. (2014)^79^ discovered that more exploratory males had more breeding attempts and raised more nestlings than less exploratory males in zebra finches. Similarly, Roth et al. (2021)^80^ observed in red junglefowl, *Gallus gallus*, that males with higher exploratory speed had greater breeding success.

### Personality expression linked to individualized prey life stage preference

Regression analyses revealed few overarching but many sub-group-specific positive correlations between activity and relative prey life stage preference, which was primarily the case in the parental plus early-life and parental experiments. Across experiments and sub-groups, consistently more active personalities had a stronger preference for mobile prey than consistently little active personalities. However, similar to personality expression, there was no single critical driver (species, sex, or prey life stage experience) of this correlation but it was determined by diverse combinations of species, sex, and prey life stage experience. If any general trend, then it was mainly females and prey eggs-experienced individuals where activity scores scaled positively with Manly’s β, though in the parental plus early-life experiment, mobile prey-experienced individuals had an increased mobile prey preference. The link between exploration propensity and prey life stage preference was less conspicuous. We detected only one sub-group where personality in exploration was predictive of their foraging preference, that is, more exploratory *P. macropilis* females with parental plus personal prey eggs experience had a stronger preference for eggs. Nonetheless, in the parental experiment there were two sub-groups with dome-shaped relations, indicating that consistently little or highly exploratory types had a stronger egg preference than flexible types. Dome-shaped relations were also found between activity scores and prey life stage preference in four sub-groups, indicating a similar trend for the distinction between consistent and flexible types. In any case, highly sophisticated and subtle differences within the same prey species lead to co-variation of the predators’ personalities, individualized foraging phenotypes and realized niche use. These links should ultimately benefit living together and mitigate inter-individual conflicts within local groups. Similarly, Herath et al. (2021)^32^ concluded that correlation between animal personality and individual specialization in diet niches should minimize intraspecific competition in common brushtail possums.

### Conclusions

Parental effects and early life experiences often interact with each other and may co-shape animal personality.^3^^,^^11^ However, studies targeting the induction or modulation of animal personality by environmental conditions rarely compared the separate and combined effects of parental and early-life experiences on personality formation later in life and typically compared stressful and benign dietary conditions (nutritional deficiencies or restricted diet). Our study provides novel insights into the role played by parental and early-life experiences, separately and together, which are not caused by major nutritional differences, in the formation of animal personality. Previous studies on nutrition-based changes in foraging behavior and personalities causally related the changes to nutritional imbalances or deficiencies and associated pleiotropic effects.^30^ These were not causal effects in our study because both types of prey life stages used in our experiments, eggs and mobile immatures, are similar in nutritional value, notwithstanding fine-scale differences in micro-nutrients due to metabolic changes associated with immature development. The most conspicuous difference is being movable versus being stationary, which requires major foraging differences in searching and handling, including prey capture and rupture.

### Limitations of the study

Our study documents plastic shifts in mean behavioral trait expression as well as behavioral repeatability of adult predatory mites following parental and/or early-life experience of different prey life stages. Regarding parental effects, there were clear maternal effects but the experimental design did not allow to ascertain a contributing role, if any, of paternal effects. Similarly, future studies should address whether early-life effects induced by a given prey life stage experience can be influenced by the prey life stage experience of the mates of the target individuals.

## Resource availability

### Lead contact

Further information and requests for resources and reagents should be directed to and will be fulfilled by the lead contact, Peter Schausberger (peter.schausberger@univie.ac.at).

### Materials availability

This study did not generate new unique reagents.

### Data and code availability

• All data reported in this paper will be shared by the lead contact upon request.
• This paper does not report original code.
• Any additional information required to reanalyze the data reported in this paper is available from the lead contact upon request.

## Acknowledgments

This study was funded by the 10.13039/501100002428Austrian Science Fund (FWF; P33787-B to P.S.). P.S. wrote part of the manuscript during an invited professorship at the University of Kyoto, which funding is greatly appreciated. We thank Mustafa Altintas for help in taking care of the rearing of the mites, and Haralabos Tsolakis, University of Palermo, and Raphael de Campos Castilho, University of Sao Paulo, for sending field-collected specimens of *P. persimilis* and *P. macropilis,* which were used as founders of our laboratory populations.

## Author contributions

P.S. conceived the study idea and acquired funding; P.S. and T.H.N. designed the experiments and analyzed the data; T.H.N. conducted the experiments, took care of the rearing and wrote the first draft of the manuscript; P.S. and T.H.N. contributed to revision, read the final version of the manuscript and approved its submission.

## Declaration of interests

The authors declare no competing interests.

## STAR★Methods

### Key resources table

**Table:** 

REAGENT or RESOURCE	SOURCE	IDENTIFIER
**Experimental models: Organisms/strains**		

*Phytoseiulus persimilis*	Collected on open field aubergine in Sicily (this paper)	N/A
*Phytoseiulus macropilis*	Collected in the field in the state of Sao Paulo, Brazil (this paper)	N/A
*Tetranychus urticae* (green form)	Collected on bean plants in a greenhouse in Vienna, Austria (this paper)	N/A
*Phaseolus vulgaris* var. Maxi BIO	Austrosaat	https://www.austrosaat.at/

**Software and algorithms**		

IBM SPSS version 29.0.1.0	IBM, Armonk, NY, USA	N/A
R software ver. 4.2.0	RCore Team (2022)^81^	https://www.R-project.org
RStudio	RStudio Team (2022)^82^	http://www.rstudio.com/
*ggplot2* package	Wickham, H. (2016)^83^	https://doi.org/10.1007/978-3-319-24277-4
*irr* package	Gamer, M., Lemon, J., & Singh, I. (2019)^84^	https://cran.rproject.org/web/packages/irr/index.html
*lme4* package	Bates, D., Mächler, M., Bolker, B., & Walker, S. (2015)^85^	https://doi.org/10.18637/jss.v067.i01

### Experimental model and subject details

#### Predatory mite origin and rearing

Predatory mites *Phytoseiulus persimilis* and *P. macropilis* used in experiments were randomly collected from populations reared separately in the laboratory in heaps of detached bean leaves infested by *T. urticae*, placed on acrylic tiles (15 x 15 x 5 cm), which edges were covered by moist tissue paper, inside a plastic box (20 x 20 x 5 cm) half-filled with tap water. Specimens of *P. persimilis* and *P. macropilis* serving as founders of the laboratory populations were field-collected in Sicily, Italy, and Sao Paulo, Brazil, respectively. The predatory mite populations were fed by adding detached leaves infested by *T. urticae* to the rearing arenas twice a week. *Tetranychus urticae* was reared on whole common bean plants, *Phaseolus vulgaris* L. The rearing units of the predatory mites and spider mites were stored in an air-conditioned room (23 ± 1°C), 60 ± 5% relative humidity (RH) and a 16:8 h L:D photoperiod, maintained by LED grow lights (SANlight FLEX 20).

### Method details

#### Parental and early-life treatments

To evaluate parental effects, early-life effects and their combination (parental plus early-life effects), we used individuals that emerged from parents that had been fed on either eggs or mobile immature stages of two-spotted spider mites *T. urticae* (parental), or which personally experienced early in life, throughout juvenile development, the spider mite prey either as eggs or mobile immature stages (early-life) or both (parental plus early-life) (Figure 1). The three experiments on (i) parental plus early life effects, (ii) only early life effects and (iii) only parental effects were run separately. In each experiment, both species, both sexes and both prey life stage treatments, eggs and mobile immatures as prey, were tested in parallel.

To induce parental effects, 10 gravid females each of *P. persimilis* and *P. macropilis* were randomly collected from their rearing units and placed in separate detached leaf arenas harboring spider mites (Figure 1). Each leaf arena consisted of a detached primary bean leaf placed adaxial side down on moist filter paper on top of a water-soaked foam block (14 x 14 x 5 cm), kept in a plastic box (20 x 20 x 6 cm) half-filled with tap water, and delimited by tissue paper wrapped around the edges of the leaf. Two-spotted spider mites were provided by brushing them from infested bean leaves, using a mite-brushing machine (BioQuip, CA, USA), onto a circular glass plate and from there into the leaf arena using a brush. Predatory mites were maintained in those arenas for several days to collect eggs (<24 h old) once per day to become the parents of the experimental animals. The eggs of each predatory mite species were randomly assigned to one of two separate small leaf arenas (dubbed parental arenas), which harbored either ∼200 eggs (<24 h old) or ∼200 mobile immatures (larvae and protonymphs) of spider mites. Each parental arena consisted of a trifoliate bean leaflet resting adaxial side down on a moist filter paper on top of a water-soaked foam block (6 x 6 x 5 cm), kept in a plastic box (10 x 10 x 6 cm) half-filled with tap water. The predatory mites were fed *ad libitum* according to their prey treatment (either eggs or mobiles of spider mites). They were allowed to develop in the arenas until they were adult and mated and had started oviposition. Parental arenas were inspected daily to record the developmental progress of the predators, and, if needed (i.e., before the spider mite eggs hatched or if the mobile immatures became too few), to transfer the predators to new prey eggs or mobile prey arenas. Eggs produced by adult females in the parental arenas described above were used in the experiments on parental effects and parental plus early-life effects.

To induce early-life effects (Figure 1), freshly laid eggs (<24 h old) of *P. persimilis* and *P. macropilis*, to become the experimental animals, were randomly assigned (15 eggs per arena) to separate small leaf arenas (constructed as the parental arenas), each of which harbored either ∼200 eggs or ∼200 mobile immatures (larvae and protonymphs) of spider mites. The predators were left to develop in these arenas until they became adult and were mated. During development, the predators were transferred to new egg or mobile arenas (according to their prey treatment) every 4 to 5 days. Mated females and males, at 2 to 3 days post maturation, were used as experimental animals.

For the experiment targeting parental plus early life effects, the experimental animals were generated by following both procedures described above, that is, both parents and the experimental animals themselves experienced exclusively either eggs or mobiles of spider mites before being subjected to the behavioral assays (Figure 1). For the experiment targeting only parental effects, the parents of the experimental animals were subjected to the parental experience procedure described above, but the experimental animals themselves were reared from the egg stage until adult in leaf arenas harboring mixed life stages of spider mites (eggs and mobiles). For the experiment targeting only early-life effects, the experimental animals were produced by parents fed mixed stages (eggs and mobiles) of spider mites in the parental arenas, and were otherwise generated by the early-life effects procedure described above (Figure 1). All leaf arenas were kept in an environmental chamber (Panasonic MLR-352H-PE) set at 23 ± 1°C, 60 ± 5% relative humidity RH, and 16:8 L:D photoperiod.

#### Behavioral assays

The behavioral assays aimed at determining the influence of the parental and early-life prey treatments (separate and in combination) on the prey life stage preference of the predators and the personality traits activity and exploration. In each experiment (subsequently called parental plus early life, early-life, and parental experiment), each gravid female and each adult male from both predator species and from each prey life stage treatment was subjected to a battery of three tests over four days (Figure 1). The number of eggs laid by the experimental females was recorded throughout the 4-d sequence of the behavioral assays.

In the first assay on day 1, each predator was given a choice between distinct eggs- and mobiles prey-patches offered on two leaf discs connected by a wax bridge. The circular leaf discs (1.5 cm Ø) were punched out from trifoliate bean leaves, using a cork borer, and placed adaxial side down on moist filter paper resting on a water-saturated foam block (6 x 6 x 5 cm), covered by tissue paper on the edges, inside a plastic box (10 x 10 x 6 cm) half-filled with tap water. The leaf discs were connected by a T-shaped wax bridge (3 cm length) built by dripping hot wax from a non-fragrant candle in between the discs onto the surface of the filter paper. One leaf disc harbored a prey patch consisting of 20 spider mite eggs while the other disc harbored 20 mobile immature spider mites. The choice arenas were monitored in 1 h intervals over 6 h and again after 24 h to record the patch residence (in egg or mobile prey patch), the number of prey items eaten, and the activity (stationary or moving) of the predators. The predators were left in the two-patch choice arenas before the second test was carried out on day 3.

In the second assay, each predator was allowed to forage on a single leaf disc harboring a single patch composed of equal proportions of mixed life stages of spider mites (20 eggs and 20 mobiles). After transfer from the two-patch choice arena, each predator was monitored in 40 min intervals over 200 min in total for their activity (stationary or moving) and the number of prey items eaten. The predators were left on the single leaf disc used in the second assay until the third assay was carried out on day 4.

In the third assay, exploration of a novel environment by the predators was assessed by releasing the predators individually inside an empty T-shaped maze cut into an acrylic plate (80 mm long × 35 mm width × 3 mm high). The maze was closed with fine mesh at the bottom and a removable microscope slide fixed by foldback clips on the upper side. Each maze consisted of two large circular cavities (15 mm Ø x 3 mm high), located at either end of the crossbar of the T, connected by an aisle (20 mm width) leading to a small cavity at the bottom end of the T, used as release site of the predators.^86^ Each of the two large cavities, the small release cavity, and the connecting aisle were considered four distinct exploration sites. After release of the predators, the predators were continuously monitored over 5 min and the frequency of exploration sites actively visited by the predators was counted.

All three tests were conducted in an air-conditioned laboratory at 23 ± 1°C, 50 ± 5% RH and natural daylight, using a Leica M80 stereo-microscope. In between assays, the experimental units were stored in an environmental chamber (Panasonic MLR-352H-PE, Japan) at 23 ± 1°C, 60 ± 5% RH, and 16:8 h L:D. Total sample sizes were 68, 48, and 47 *P. persimilis* and 68, 50, and 43 *P. macropilis* in the parental plus early-life, parental and early-life experiments. Each experimental animal and each experimental patch arrangement and acrylic T-maze were used only once in each assay.

### Quantification and statistical analysis

Statistical analysis was performed using R software ver. 4.2.0^81^ and RStudio^82^ (Data S1 for codes) and IBM SPSS Statistics ver. 29.0.1.0. Manly’s preference index β^87^ was used to quantify the prey life stage preference of the predators from the number of prey eggs and mobile prey consumed in the first assay (two-patch choice) using the following equation:

$$\beta =\frac{\log\left(\frac{R}{r}\right)}{\log\left(\frac{B}{b}\right)+\log\left(\frac{R}{r}\right)}$$

where ß = Manly’s preference index for diet R; R and r and B and b indicate the number of prey R (mobile spider mites) and B (spider mite eggs) alive at the assay’s start and end. In this two-prey types combination, β = 0.5 indicates no preference, β > 0.5 indicates a preference for prey R (mobile spider mites), and β < 0.5 indicates a preference for prey B (spider mite eggs).

In each experiment (parental plus early-life, early-life, parental), separate generalized linear models (GLMs) were used to analyze the influence of prey life stage experience, species and sex on mean Manly’s β (normal, identity link), mean activity (proportion moving; normal, identity link), and mean exploration (number of disc changes; Poisson, logit link) in the first (two-patch choice) assay and total prey consumption (normal, identity link) across all assays. GLM was also used to analyze the influence of prey life stage experience and species on the total number of eggs laid by each female (normal, identity link) across all three assays. To obtain the most parsimonious model in each analysis, we started with the full model and removed non-significant interaction terms. Models were built in the R environment using the *lme4* package.^85^

To assess the predators’ personalities in activity and exploration, we calculated the intraclass correlation coefficients (ICC; two-way-random, consistency, average measure), also called repeatability, using the *irr* package.^84^ For activity, we first calculated an average activity score (proportion of time moving) for each individual in the first and second assay. For exploration, we used the number of leaf disc changes in the first assay and the number of sites visited in the third assay (square root-transformed before analysis). In each experiment, ICCs for activity and exploration were calculated at several levels: in total, at intermediate levels, i.e., for each predator species, for each prey treatment, and for each sex, and the combinations of these factors.

In each experiment (parental plus early-life, early-life, parental), separate GLMs (Poisson, logit link) were used to evaluate the within-group composition of personality types,^88^ as influenced by species, prey life stage experience and sex. To this end, we first categorized the personality types in activity based on the consistency and level of the proportional time moving in the first and second assay, and the personality types in exploration based on the consistency and level in the number of changes between leaf discs in the first assay and the number of sites visited in the third assay (Table S1 for categorization of personality types).

To assess the fitness implications of having a given personality type in activity and exploration, we ran linear regressions of the number of eggs produced by each predatory mite female during the behavioral assays on the personality scores in activity and exploration. To scrutinize the links between personality types and prey life stage preference, we conducted linear and quadratic regressions of the personality scores in activity and exploration on Manly’s β. In each experiment, each regression was run across subgroups defined by species, sex and prey treatment, within each subgroup and at all intermediate grouping levels. The best fitting models were determined by curve estimation using IBM SPSS Statistics ver. 29.0.1.0. Figures 2, 3, 4, 5, 6, 7, and 8 were created by the ggplot2 package^83^ in R software ver. 4.2.0^81^ and RStudio.^82^
