# Supplementary material for: Parental and personal experience drive personality formation and individual niche diversification in group-living mites
Source: iScience. 2025 Apr 14;28(5):112424. doi: 10.1016/j.isci.2025.112424 (PMC12059715; doi:10.1016/j.isci.2025.112424)
Supplement: Document S1. Table S1 and Data S1 [file mmc1.pdf]

iScience, Volume *28*

Supplemental information

Parental and personal experience drive personality formation and individual niche diversification in group-living mites

Thi Hanh Nguyen and Peter Schausberger

Table S 1. Categorization of personality types in activity and exploration, judged from the performance and consistency in two assays each per behavioral trait; related to figures 5-8.

**Activity**		**Proportion of time moving in assays 1 and 2**[a]
	0	≤0.33, ≤0.33
	1	≤0.33, 0.67>x>0.33
	2	0.67>x>0.33, 0.67≥x≥0.33
	3	≤0.33, ≥0.67
	4	0.67>x>0.33, ≥0.67
	5	≥0.67, ≥0.67
**Exploration**		**Number of exploratory events in assays 1 and 3**[b]
	0	$x_1 \leq 3$, $x_2 \leq 11$
	1	$x_1 \leq 3$, $23 > x_2 > 11$/$6 > x_1 > 3$, $x_2 \leq 11$
	2	$6 > x_1 > 3$,$23 > x_2 > 11$
	3	$x_1 \leq 3$, $x_2 \geq 23$/ $x_1 \geq 7$, $x_2 \leq 11$
	4	$6 > x_1 > 3$, $x_2 \geq 23$/ $x_1 \geq 7$, $23 > x_2 > 11$
	5	$x_1 \geq 7$, $x_2 \geq 23$

[a]The sequence of assays is irrelevant.

[b]The number of changes between leaf discs in assay 1 (x_1) ranged from 0 to 9, which were allocated to three sections (0-3, 4-6, 7-9); the number of places visited in assay 3 (x_2) ranged from 0 to 36, which were allocated to three sections (0-11, 12-23, 24-36).

Data S1. R-codes used in statistical analyses; related to figures 2-8 and table 1

PP+PM.PE

2024-12-04

Parental + early life effects

Dataset

```
A.PE <- read.csv("Activity.PE.old.csv", header=TRUE)
A.PE.2 <- subset(A.PE, sex%in% "female")
A.PE.3 <- subset(A.PE, species%in% "persimilis" & sex%in% "female"&
treatment%in% "egg")
A.PE.4 <- subset(A.PE, species%in% "persimilis" & sex%in% "male"&
treatment%in% "egg")
A.PE.5 <- subset(A.PE, species%in% "macropilis" & sex%in% "female"&
treatment%in% "egg")
A.PE.6 <- subset(A.PE, species%in% "macropilis" & sex%in% "male"&
treatment%in% "egg")
A.PE.7 <- subset(A.PE, species%in% "macropilis"& sex%in% "male"&
treatment%in% "mobile")
A.PE.8 <- subset(A.PE, species%in% "persimilis" & sex%in% "female"&
treatment%in% "mobile")
A.PE.9 <- subset(A.PE, species%in% "persimilis" & sex%in% "male"&
treatment%in% "mobile")
A.PE.10 <- subset(A.PE, species%in% "macropilis" & sex%in% "female"&
treatment%in% "mobile")
A.PE.11 <- subset(A.PE, species%in% "macropilis")
A.PE.12 <- subset(A.PE, species%in% "persimilis")
A.PE.13 <- subset(A.PE, sex%in% "male")
A.PE.14 <- subset(A.PE, treatment%in% "egg")
A.PE.15 <- subset(A.PE, treatment%in% "mobile")

A.PE$activity_scores2<-A.PE$activity_scores^2
A.PE.2$activity_scores2<-A.PE.2$activity_scores^2
A.PE.3$activity_scores2<-A.PE.3$activity_scores^2
A.PE.4$activity_scores2<-A.PE.4$activity_scores^2
A.PE.5$activity_scores2<-A.PE.5$activity_scores^2
A.PE.6$activity_scores2<-A.PE.6$activity_scores^2
A.PE.7$activity_scores2<-A.PE.7$activity_scores^2
A.PE.8$activity_scores2<-A.PE.8$activity_scores^2
A.PE.9$activity_scores2<-A.PE.9$activity_scores^2
A.PE.10$activity_scores2<-A.PE.10$activity_scores^2
A.PE.11$activity_scores2<-A.PE.11$activity_scores^2
A.PE.12$activity_scores2<-A.PE.12$activity_scores^2
A.PE.13$activity_scores2<-A.PE.13$activity_scores^2
A.PE.14$activity_scores2<-A.PE.14$activity_scores^2
A.PE.15$activity_scores2<-A.PE.15$activity_scores^2

A.PE$exploration_scores2<-A.PE$exploration_scores^2
A.PE.2$exploration_scores2<-A.PE.2$exploration_scores^2
```

```
A.PE.3$exploration_scores2<-A.PE.3$exploration_scores^2
A.PE.4$exploration_scores2<-A.PE.4$exploration_scores^2
A.PE.5$exploration_scores2<-A.PE.5$exploration_scores^2
A.PE.6$exploration_scores2<-A.PE.6$exploration_scores^2
A.PE.7$exploration_scores2<-A.PE.7$exploration_scores^2
A.PE.8$exploration_scores2<-A.PE.8$exploration_scores^2
A.PE.9$exploration_scores2<-A.PE.9$exploration_scores^2
A.PE.10$exploration_scores2<-A.PE.10$exploration_scores^2
A.PE.11$exploration_scores2<-A.PE.11$exploration_scores^2
A.PE.12$exploration_scores2<-A.PE.12$exploration_scores^2
A.PE.13$exploration_scores2<-A.PE.13$exploration_scores^2
A.PE.14$exploration_scores2<-A.PE.14$exploration_scores^2
A.PE.15$exploration_scores2<-A.PE.15$exploration_scores^2
```

Part 1: Population mean

Manly's index in the first assay:

```
manly.1.end<-glm(Manly_index_1~treatment*species*sex,data = A.PE, family
=gaussian(link=identity))
Anova(manly.1.end, type=3)
```

Remove non significant interactions:

```
manly.1.end.1<-glm(Manly_index_1~treatment+species+sex,data = A.PE, family
=gaussian(link=identity))
Anova(manly.1.end.1, type=3)
```

Mean activity in the first assay:

```
activity.1<-glm(proportion_moving_1~treatment*species*sex, data=A.PE,family =
gaussian(link=identity))
Anova(activity.1, type=3)
```

Remove non significant interactions:

```
activity.2<-glm(proportion_moving_1~treatment+species+sex, data=A.PE, family
= gaussian(link=identity))
Anova(activity.2, type=3)
```

Mean exploration in the first assay:

```
exploration.2<-glm(no_changes~treatment*species*sex-treatment:species:sex,
data=A.PE, family = poisson(link = "log"))
Anova(exploration.2,type=3)
```

Remove non significant interactions:

```
exploration.3<-glm(no_changes~treatment+species+sex, data=A.PE, family =
poisson(link = "log"))
Anova(exploration.3,type=3)
```

Total prey consumption in the first two assays:

```
m5.2<- glm(prey_eaten ~ treatment*species*-treatment:species,data=A.PE,
family=gaussian(link=identity))
Anova(m5.2, type=3)
```

Remove non significant interactions:

```
m5.3<- glm(prey_eaten ~ treatment+species+sex+species:sex, data=A.PE,
family=gaussian(link=identity))
Anova(m5.3, type=3)
```

Total egg laying:

```
egg.laying <- glm(total_egg ~ treatment*species, data=A.PE.2,family =
gaussian(link=identity))
Anova(egg.laying, type=3)
```

Remove non significant interactions:

```
egg.laying <- glm(total_egg ~ treatment+species, data=A.PE.2,family =
gaussian(link=identity))
Anova(egg.laying, type=3)
```

Part 2: Personality expression (Intraclass correlation coefficients - ICCs)

Personality in activity:

```
R.1<-icc(cbind(A.PE$proportion_moving_1,A.PE$proportion_moving_2),model =
"twoway", unit = "average")
print(R.1)
R.2<-icc(cbind(A.PE.2$proportion_moving_1,A.PE.2$proportion_moving_2),model =
"twoway", unit = "average")
print(R.2)
R.3<-icc(cbind(A.PE.3$proportion_moving_1,A.PE.3$proportion_moving_2),model =
"twoway", unit = "average")
print(R.3)
R.4<-icc(cbind(A.PE.4$proportion_moving_1,A.PE.4$proportion_moving_2),model =
"twoway", unit = "average")
print(R.4)
R.5<-icc(cbind(A.PE.5$proportion_moving_1,A.PE.5$proportion_moving_2),model =
"twoway", unit = "average")
print(R.5)
R.6<-icc(cbind(A.PE.6$proportion_moving_1,A.PE.6$proportion_moving_2),model =
"twoway", unit = "average")
print(R.6)
R.7<-icc(cbind(A.PE.7$proportion_moving_1,A.PE.7$proportion_moving_2),model =
"twoway", unit = "average")
print(R.7)
R.8<-icc(cbind(A.PE.8$proportion_moving_1,A.PE.8$proportion_moving_2),model =
"twoway", unit = "average")
```

```
print(R.8)
R.9<-icc(cbind(A.PE.9$proportion_moving_1,A.PE.9$proportion_moving_2),model =
"twoway", unit = "average")
print(R.9)
R.10<-
icc(cbind(A.PE.10$proportion_moving_1,A.PE.10$proportion_moving_2),model =
"twoway", unit = "average")
print(R.10)
R.11<-
icc(cbind(A.PE.11$proportion_moving_1,A.PE.11$proportion_moving_2),model =
"twoway", unit = "average")
print(R.11)
R.12<-
icc(cbind(A.PE.12$proportion_moving_1,A.PE.12$proportion_moving_2),model =
"twoway", unit = "average")
print(R.12)
R.13<-
icc(cbind(A.PE.13$proportion_moving_1,A.PE.13$proportion_moving_2),model =
"twoway", unit = "average")
print(R.13)
R.14<-
icc(cbind(A.PE.14$proportion_moving_1,A.PE.14$proportion_moving_2),model =
"twoway", unit = "average")
print(R.14)
R.15<-
icc(cbind(A.PE.15$proportion_moving_1,A.PE.15$proportion_moving_2),model =
"twoway", unit = "average")
print(R.15)
```

Personality in exploration:

```
R.1<-icc(cbind(A.PE$no_changes,sqrt(A.PE$no_places_3)),model = "twoway", unit
= "average")
print(R.1)
R.2<-icc(cbind(A.PE.2$no_changes,sqrt(A.PE.2$no_places_3)),model = "twoway",
unit = "average")
print(R.2)
R.3<-icc(cbind(A.PE.3$no_changes,sqrt(A.PE.3$no_places_3)),model = "twoway",
unit = "average")
print(R.3)
R.4<-icc(cbind(A.PE.4$no_changes,sqrt(A.PE.4$no_places_3)),model = "twoway",
unit = "average")
print(R.4)
R.5<-icc(cbind(A.PE.5$no_changes,sqrt(A.PE.5$no_places_3)),model = "twoway",
unit = "average")
print(R.5)
R.6<-icc(cbind(A.PE.6$no_changes,sqrt(A.PE.6$no_places_3)),model = "twoway",
unit = "average")
print(R.6)
R.7<-icc(cbind(A.PE.7$no_changes,sqrt(A.PE.7$no_places_3)),model = "twoway",
unit = "average")
```

```
print(R.7)
R.8<-icc(cbind(A.PE.8$no_changes,sqrt(A.PE.8$no_places_3)),model = "twoway",
unit = "average")
print(R.8)
R.9<-icc(cbind(A.PE.9$no_changes,sqrt(A.PE.9$no_places_3)),model = "twoway",
unit = "average")
print(R.9)
R.10<-icc(cbind(A.PE.10$no_changes,sqrt(A.PE.10$no_places_3)),model =
"twoway", unit = "average")
print(R.10)
R.11<-icc(cbind(A.PE.11$no_changes,sqrt(A.PE.11$no_places_3)),model =
"twoway", unit = "average")
print(R.11)
R.12<-icc(cbind(A.PE.12$no_changes,sqrt(A.PE.12$no_places_3)),model =
"twoway", unit = "average")
print(R.12)
R.13<-icc(cbind(A.PE.13$no_changes,sqrt(A.PE.13$no_places_3)),model =
"twoway", unit = "average")
print(R.13)
R.14<-icc(cbind(A.PE.14$no_changes,sqrt(A.PE.14$no_places_3)),model =
"twoway", unit = "average")
print(R.14)
R.15<-icc(cbind(A.PE.15$no_changes,sqrt(A.PE.15$no_places_3)),model =
"twoway", unit = "average")
print(R.15)
```

Part 3: Personality composition

Personality composition in activity:

```
activity.1<-glm(activity_scores~treatment*species*sex,data = A.PE, family
=poisson(link=log))
Anova(activity.1, type = 3 )
```

Remove non significant interactions:

```
activity.2.2<-glm(activity_scores~treatment+species+sex,data = A.PE, family
=poisson(link=log))
Anova(activity.2.2, type=3)
```

Personality composition in exploration:

```
ex.1.1<-glm(exploration_scores~treatment*species*sex,data = A.PE, family
=poisson(link=log))
Anova(ex.1.1,type=3 )
```

Remove non significant interactions:

```
ex.2.1<-glm(exploration_scores~treatment+species+sex,data = A.PE, family
=poisson(link=log))
Anova(ex.2.1,type=3 )
```

Part 4: Personality types and short-term fitness

Activity:

```
linearmodel<-lm(total_egg~activity_scores, data = A.PE.2)
Anova(linearmodel, type = 3)
linearmodel<-lm(total_egg~activity_scores, data = A.PE.3)
Anova(linearmodel, type = 3)
linearmodel<-lm(total_egg~activity_scores, data = A.PE.5)
Anova(linearmodel, type = 3)
linearmodel<-lm(total_egg~activity_scores, data = A.PE.8)
Anova(linearmodel, type = 3)
linearmodel<-lm(total_egg~activity_scores, data = A.PE.10)
Anova(linearmodel, type = 3)
linearmodel<-lm(total_egg~activity_scores, data = A.PE.11)
Anova(linearmodel, type = 3)
linearmodel<-lm(total_egg~activity_scores, data = A.PE.12)
Anova(linearmodel, type = 3)
```

Exploration:

```
linearmodel<-lm(total_egg~exploration_scores, data = A.PE.2)
Anova(linearmodel, type = 3)
linearmodel<-lm(total_egg~exploration_scores, data = A.PE.3)
Anova(linearmodel, type = 3)
linearmodel<-lm(total_egg~exploration_scores, data = A.PE.5)
Anova(linearmodel, type = 3)
linearmodel<-lm(total_egg~exploration_scores, data = A.PE.8)
Anova(linearmodel, type = 3)
linearmodel<-lm(total_egg~exploration_scores, data = A.PE.10)
Anova(linearmodel, type = 3)
linearmodel<-lm(total_egg~exploration_scores, data = A.PE.11)
Anova(linearmodel, type = 3)
linearmodel<-lm(total_egg~exploration_scores, data = A.PE.12)
Anova(linearmodel, type = 3)
```

Part 5: Personality types and prey stage preference (Manly index)

Pearson correlations:

Activity:

```
linearmodel<-lm(Manly_index_1~activity_scores, data = A.PE)
Anova(linearmodel, type = 3)
linearmodel<-lm(Manly_index_1~activity_scores, data = A.PE.2)
Anova(linearmodel, type = 3)
linearmodel<-lm(Manly_index_1~activity_scores, data = A.PE.3)
Anova(linearmodel, type = 3)
linearmodel<-lm(Manly_index_1~activity_scores, data = A.PE.4)
```

```
Anova(linearmodel, type = 3)
linearmodel<-lm(Manly_index_1~activity_scores, data = A.PE.5)
Anova(linearmodel, type = 3)
linearmodel<-lm(Manly_index_1~activity_scores, data = A.PE.6)
Anova(linearmodel, type = 3)
linearmodel<-lm(Manly_index_1~activity_scores, data = A.PE.7)
Anova(linearmodel, type = 3)
linearmodel<-lm(Manly_index_1~activity_scores, data = A.PE.8)
Anova(linearmodel, type = 3)
linearmodel<-lm(Manly_index_1~activity_scores, data = A.PE.9)
Anova(linearmodel, type = 3)
linearmodel<-lm(Manly_index_1~activity_scores, data = A.PE.10)
Anova(linearmodel, type = 3)
linearmodel<-lm(Manly_index_1~activity_scores, data = A.PE.11)
Anova(linearmodel, type = 3)
linearmodel<-lm(Manly_index_1~activity_scores, data = A.PE.12)
Anova(linearmodel, type = 3)
linearmodel<-lm(Manly_index_1~activity_scores, data = A.PE.13)
Anova(linearmodel, type = 3)
linearmodel<-lm(Manly_index_1~activity_scores, data = A.PE.14)
Anova(linearmodel, type = 3)
linearmodel<-lm(Manly_index_1~activity_scores, data = A.PE.15)
Anova(linearmodel, type = 3)
```

Exploration:

```
linearmodel<-lm(Manly_index_1~exploration_scores, data = A.PE)
Anova(linearmodel, type = 3)
linearmodel<-lm(Manly_index_1~exploration_scores, data = A.PE.2)
Anova(linearmodel, type = 3)
linearmodel<-lm(Manly_index_1~exploration_scores, data = A.PE.3)
Anova(linearmodel, type = 3)
linearmodel<-lm(Manly_index_1~exploration_scores, data = A.PE.4)
Anova(linearmodel, type = 3)
linearmodel<-lm(Manly_index_1~exploration_scores, data = A.PE.5)
Anova(linearmodel, type = 3)
linearmodel<-lm(Manly_index_1~exploration_scores, data = A.PE.6)
Anova(linearmodel, type = 3)
linearmodel<-lm(Manly_index_1~exploration_scores, data = A.PE.7)
Anova(linearmodel, type = 3)
linearmodel<-lm(Manly_index_1~exploration_scores, data = A.PE.8)
Anova(linearmodel, type = 3)
linearmodel<-lm(Manly_index_1~exploration_scores, data = A.PE.9)
Anova(linearmodel, type = 3)
linearmodel<-lm(Manly_index_1~exploration_scores, data = A.PE.10)
Anova(linearmodel, type = 3)
linearmodel<-lm(Manly_index_1~exploration_scores, data = A.PE.11)
Anova(linearmodel, type = 3)
linearmodel<-lm(Manly_index_1~exploration_scores, data = A.PE.12)
Anova(linearmodel, type = 3)
linearmodel<-lm(Manly_index_1~exploration_scores, data = A.PE.13)
Anova(linearmodel, type = 3)
```

```
linearmodel<-lm(Manly_index_1~exploration_scores, data = A.PE.14)
Anova(linearmodel, type = 3)
linearmodel<-lm(Manly_index_1~exploration_scores, data = A.PE.15)
Anova(linearmodel, type = 3)
```

Quadratic regression:

Activity:

```
quadraticmodel<-lm(Manly_index_1~activity_scores+activity_scores2, data =
A.PE)
Anova(quadraticmodel, type = 3)
quadraticmodel<-lm(Manly_index_1~activity_scores+activity_scores2, data =
A.PE.2)
Anova(quadraticmodel, type = 3)
quadraticmodel<-lm(Manly_index_1~activity_scores+activity_scores2, data =
A.PE.3)
Anova(quadraticmodel, type = 3)
quadraticmodel<-lm(Manly_index_1~activity_scores+activity_scores2, data =
A.PE.4)
Anova(quadraticmodel, type = 3)
quadraticmodel<-lm(Manly_index_1~activity_scores+activity_scores2, data =
A.PE.5)
Anova(quadraticmodel, type = 3)
quadraticmodel<-lm(Manly_index_1~activity_scores+activity_scores2, data =
A.PE.6)
Anova(quadraticmodel, type = 3)
quadraticmodel<-lm(Manly_index_1~activity_scores+activity_scores2, data =
A.PE.7)
Anova(quadraticmodel, type = 3)
quadraticmodel<-lm(Manly_index_1~activity_scores+activity_scores2, data =
A.PE.8)
Anova(quadraticmodel, type = 3)
quadraticmodel<-lm(Manly_index_1~activity_scores+activity_scores2, data =
A.PE.9)
Anova(quadraticmodel, type = 3)
quadraticmodel<-lm(Manly_index_1~activity_scores+activity_scores2, data =
A.PE.10)
Anova(quadraticmodel, type = 3)
quadraticmodel<-lm(Manly_index_1~activity_scores+activity_scores2, data =
A.PE.11)
Anova(quadraticmodel, type = 3)
quadraticmodel<-lm(Manly_index_1~activity_scores+activity_scores2, data =
A.PE.12)
Anova(quadraticmodel, type = 3)
quadraticmodel<-lm(Manly_index_1~activity_scores+activity_scores2, data =
A.PE.13)
Anova(quadraticmodel, type = 3)
quadraticmodel<-lm(Manly_index_1~activity_scores+activity_scores2, data =
A.PE.14)
Anova(quadraticmodel, type = 3)
quadraticmodel<-lm(Manly_index_1~activity_scores+activity_scores2, data =
```

```
A.PE.15)
Anova(quadraticmodel, type = 3)
```

Exploration:

```
quadraticmodel.1<-lm(Manly_index_1~exploration_scores+exploration_scores2,
data = A.PE)
Anova(quadraticmodel.1, type = 3)
quadraticmodel.1<-lm(Manly_index_1~exploration_scores+exploration_scores2,
data = A.PE.2)
Anova(quadraticmodel.1, type = 3)
quadraticmodel.1<-lm(Manly_index_1~exploration_scores+exploration_scores2,
data = A.PE.3)
Anova(quadraticmodel.1, type = 3)
quadraticmodel.1<-lm(Manly_index_1~exploration_scores+exploration_scores2,
data = A.PE.5)
Anova(quadraticmodel.1, type = 3)
quadraticmodel.1<-lm(Manly_index_1~exploration_scores+exploration_scores2,
data = A.PE.8)
Anova(quadraticmodel.1, type = 3)
quadraticmodel.1<-lm(Manly_index_1~exploration_scores+exploration_scores2,
data = A.PE.9)
Anova(quadraticmodel.1, type = 3)
quadraticmodel.1<-lm(Manly_index_1~exploration_scores+exploration_scores2,
data = A.PE.10)
Anova(quadraticmodel.1, type = 3)
quadraticmodel.1<-lm(Manly_index_1~exploration_scores+exploration_scores2,
data = A.PE.11)
Anova(quadraticmodel.1, type = 3)
quadraticmodel.1<-lm(Manly_index_1~exploration_scores+exploration_scores2,
data = A.PE.12)
Anova(quadraticmodel.1, type = 3)
quadraticmodel.1<-lm(Manly_index_1~exploration_scores+exploration_scores2,
data = A.PE.13)
Anova(quadraticmodel.1, type = 3)
quadraticmodel.1<-lm(Manly_index_1~exploration_scores+exploration_scores2,
data = A.PE.14)
Anova(quadraticmodel.1, type = 3)
quadraticmodel.1<-lm(Manly_index_1~exploration_scores+exploration_scores2,
data = A.PE.15)
Anova(quadraticmodel.1, type = 3)
```

PP+PM.P

2024-12-05

Parental effects

Dataset

```
A.P <- read.csv("Activity.P.old.csv", header=TRUE)
A.P.2 <- subset(A.P, sex%in% "female")
A.P.3 <- subset(A.P, species%in% "persimilis" & sex%in% "female"&
treatment%in% "egg")
A.P.4 <- subset(A.P, species%in% "persimilis" & sex%in% "male"& treatment%in%
"egg")
A.P.5 <- subset(A.P, species%in% "macropilis" & sex%in% "female"&
treatment%in% "egg")
A.P.6 <- subset(A.P, species%in% "macropilis" & sex%in% "male"& treatment%in%
"egg")
A.P.7 <- subset(A.P, species%in% "macropilis"& sex%in% "male"& treatment%in%
"mobile")
A.P.8 <- subset(A.P, species%in% "persimilis" & sex%in% "female"&
treatment%in% "mobile")
A.P.9 <- subset(A.P, species%in% "persimilis" & sex%in% "male"& treatment%in%
"mobile")
A.P.10 <- subset(A.P, species%in% "macropilis" & sex%in% "female"&
treatment%in% "mobile")
A.P.11 <- subset(A.P, species%in% "macropilis")
A.P.12 <- subset(A.P, species%in% "persimilis")
A.P.13 <- subset(A.P, sex%in% "male")
A.P.14 <- subset(A.P,  treatment%in% "egg")
A.P.15 <- subset(A.P, treatment%in% "mobile")

A.P$activity_scores2<-A.P$activity_scores^2
A.P.2$activity_scores2<-A.P.2$activity_scores^2
A.P.3$activity_scores2<-A.P.3$activity_scores^2
A.P.4$activity_scores2<-A.P.4$activity_scores^2
A.P.5$activity_scores2<-A.P.5$activity_scores^2
A.P.6$activity_scores2<-A.P.6$activity_scores^2
A.P.7$activity_scores2<-A.P.7$activity_scores^2
A.P.8$activity_scores2<-A.P.8$activity_scores^2
A.P.9$activity_scores2<-A.P.9$activity_scores^2
A.P.10$activity_scores2<-A.P.10$activity_scores^2
A.P.11$activity_scores2<-A.P.11$activity_scores^2
A.P.12$activity_scores2<-A.P.12$activity_scores^2
A.P.13$activity_scores2<-A.P.13$activity_scores^2
A.P.14$activity_scores2<-A.P.14$activity_scores^2
A.P.15$activity_scores2<-A.P.15$activity_scores^2

A.P$exploration_scores2<-A.P$exploration_scores^2
A.P.2$exploration_scores2<-A.P.2$exploration_scores^2
```

```
A.P.3$exploration_scores2<-A.P.3$exploration_scores^2
A.P.4$exploration_scores2<-A.P.4$exploration_scores^2
A.P.5$exploration_scores2<-A.P.5$exploration_scores^2
A.P.6$exploration_scores2<-A.P.6$exploration_scores^2
A.P.7$exploration_scores2<-A.P.7$exploration_scores^2
A.P.8$exploration_scores2<-A.P.8$exploration_scores^2
A.P.9$exploration_scores2<-A.P.9$exploration_scores^2
A.P.10$exploration_scores2<-A.P.10$exploration_scores^2
A.P.11$exploration_scores2<-A.P.11$exploration_scores^2
A.P.12$exploration_scores2<-A.P.12$exploration_scores^2
A.P.13$exploration_scores2<-A.P.13$exploration_scores^2
A.P.14$exploration_scores2<-A.P.14$exploration_scores^2
A.P.15$exploration_scores2<-A.P.15$exploration_scores^2
```

Part 1: Population mean

Manly's index in the first assay:

```
manly.1.end<-glm(Manly_index_1~treatment*species*sex,data = A.P, family
=gaussian(link=identity))
Anova(manly.1.end, type=3)
```

Remove non significant interactions:

```
manly.1.end.1<-glm(Manly_index_1~treatment+species+sex+treatment:sex,data =
A.P, family =gaussian(link=identity))
Anova(manly.1.end.1, type=3)
```

Mean activity in the first assay:

```
activity.1<-glm(proportion_moving_1~treatment*species*sex-
treatment:species:sex, data=A.P, family = gaussian(link=identity))
Anova(activity.1,type=3)
```

Remove non significant interactions:

```
activity.2<-glm(proportion_moving_1~treatment+species+sex, data=A.P, family =
gaussian(link=identity))
Anova(activity.2,type=3)
```

Mean exploration in the first assay:

```
exploration.2<-glm(no_changes~treatment*species*sex-treatment:species:sex,
data=A.P, family = poisson(link = "log"))
Anova(exploration.2,type=3)
```

Remove non significant interactions:

```
exploration.1<-glm(no_changes~treatment+species+sex, data=A.P, family =
poisson(link = "log"))
Anova(exploration.1,type=3)
```

Total prey consumption in the first two assays:

```
m5.2<- glm(prey_eaten ~ treatment*species*sex-treatment:species:sex,
data=A.P, family=gaussian)
Anova(m5.2, type=3)
```

Remove non significant interactions:

```
m5.3<- glm(prey_eaten ~ treatment+species+sex+species:sex, data=A.P,
family=gaussian)
Anova(m5.3, type=3)
```

Total egg laying:

```
egg.laying <- glm(total_egg ~ treatment*species, data=A.P.2,family =
gaussian(link=identity))
Anova(egg.laying, type=3)
```

Remove non significant interactions:

```
egg.laying <- glm(total_egg ~ treatment+species, data=A.P.2,family =
gaussian(link=identity))
Anova(egg.laying, type = 3)
```

Part 2: Personality expression (Intraclass correlation coefficients - ICCs)

Personality in activity:

```
R.1<-icc(cbind(A.P$proportion_moving_1,A.P$proportion_moving_2),model =
"twoway", unit = "average")
print(R.1)
R.2<-icc(cbind(A.P.2$proportion_moving_1,A.P.2$proportion_moving_2),model =
"twoway", unit = "average")
print(R.2)
R.3<-icc(cbind(A.P.3$proportion_moving_1,A.P.3$proportion_moving_2),model =
"twoway", unit = "average")
print(R.3)
R.4<-icc(cbind(A.P.4$proportion_moving_1,A.P.4$proportion_moving_2),model =
"twoway", unit = "average")
print(R.4)
R.5<-icc(cbind(A.P.5$proportion_moving_1,A.P.5$proportion_moving_2),model =
"twoway", unit = "average")
print(R.5)
R.6<-icc(cbind(A.P.6$proportion_moving_1,A.P.6$proportion_moving_2),model =
"twoway", unit = "average")
print(R.6)
R.7<-icc(cbind(A.P.7$proportion_moving_1,A.P.7$proportion_moving_2),model =
"twoway", unit = "average")
print(R.7)
R.8<-icc(cbind(A.P.8$proportion_moving_1,A.P.8$proportion_moving_2),model =
"twoway", unit = "average")
```

```
print(R.8)
R.9<-icc(cbind(A.P.9$proportion_moving_1,A.P.9$proportion_moving_2),model =
"twoway", unit = "average")
print(R.9)
R.10<-icc(cbind(A.P.10$proportion_moving_1,A.P.10$proportion_moving_2),model
= "twoway", unit = "average")
print(R.10)
R.11<-icc(cbind(A.P.11$proportion_moving_1,A.P.11$proportion_moving_2),model
= "twoway", unit = "average")
print(R.11)
R.12<-icc(cbind(A.P.12$proportion_moving_1,A.P.12$proportion_moving_2),model
= "twoway", unit = "average")
print(R.12)
R.13<-icc(cbind(A.P.13$proportion_moving_1,A.P.13$proportion_moving_2),model
= "twoway", unit = "average")
print(R.13)
R.14<-icc(cbind(A.P.14$proportion_moving_1,A.P.14$proportion_moving_2),model
= "twoway", unit = "average")
print(R.14)
R.15<-icc(cbind(A.P.15$proportion_moving_1,A.P.15$proportion_moving_2),model
= "twoway", unit = "average")
print(R.15)
```

Personality in exploration:

```
R.1<-icc(cbind(A.P$no_changes,sqrt(A.P$no_places_3)),model = "twoway", unit =
"average")
print(R.1)
R.2<-icc(cbind(A.P.2$no_changes,sqrt(A.P.2$no_places_3)),model = "twoway",
unit = "average")
print(R.2)
R.3<-icc(cbind(A.P.3$no_changes,sqrt(A.P.3$no_places_3)),model = "twoway",
unit = "average")
print(R.3)
R.4<-icc(cbind(A.P.4$no_changes,sqrt(A.P.4$no_places_3)),model = "twoway",
unit = "average")
print(R.4)
R.5<-icc(cbind(A.P.5$no_changes,sqrt(A.P.5$no_places_3)),model = "twoway",
unit = "average")
print(R.5)
R.6<-icc(cbind(A.P.6$no_changes,sqrt(A.P.6$no_places_3)),model = "twoway",
unit = "average")
print(R.6)
R.7<-icc(cbind(A.P.7$no_changes,sqrt(A.P.7$no_places_3)),model = "twoway",
unit = "average")
print(R.7)
R.8<-icc(cbind(A.P.8$no_changes,sqrt(A.P.8$no_places_3)),model = "twoway",
unit = "average")
print(R.8)
R.9<-icc(cbind(A.P.9$no_changes,sqrt(A.P.9$no_places_3)),model = "twoway",
unit = "average")
```

```
print(R.9)
R.10<-icc(cbind(A.P.10$no_changes,sqrt(A.P.10$no_places_3)),model = "twoway",
unit = "average")
print(R.10)
R.11<-icc(cbind(A.P.11$no_changes,sqrt(A.P.11$no_places_3)),model = "twoway",
unit = "average")
print(R.11)
R.12<-icc(cbind(A.P.12$no_changes,sqrt(A.P.12$no_places_3)),model = "twoway",
unit = "average")
print(R.12)
R.13<-icc(cbind(A.P.13$no_changes,sqrt(A.P.13$no_places_3)),model = "twoway",
unit = "average")
print(R.13)
R.14<-icc(cbind(A.P.14$no_changes,sqrt(A.P.14$no_places_3)),model = "twoway",
unit = "average")
print(R.14)
R.15<-icc(cbind(A.P.15$no_changes,sqrt(A.P.15$no_places_3)),model = "twoway",
unit = "average")
print(R.15)
```

Part 3: Personality composition

Personality composition in activity:

```
activity.1<-glm(activity_scores~treatment*species*sex,data = A.P, family
=poisson(link=log))
Anova(activity.1, type = 3 )
```

Remove non significant interactions:

```
activity.2.2<-glm(activity_scores~treatment+species+sex,data = A.P, family
=poisson(link=log))
Anova(activity.2.2, type=3)
```

Personality composition in exploration:

```
ex.1.1<-glm(exploration_scores~treatment*species*sex,data = A.P, family
=poisson(link=log))
Anova(ex.1.1,type=3 )
```

Remove non significant interactions:

```
ex.2.1<-glm(exploration_scores~treatment+species+sex,data = A.P, family
=poisson(link=log))
Anova(ex.2.1,type=3 )
```

Part 4: Personality types and short-term fitness

Activity:

```
linearmodel<-lm(total_egg~activity_scores, data = A.P.2)
Anova(linearmodel, type = 3)
linearmodel<-lm(total_egg~activity_scores, data = A.P.3)
Anova(linearmodel, type = 3)
linearmodel<-lm(total_egg~activity_scores, data = A.P.5)
Anova(linearmodel, type = 3)
linearmodel<-lm(total_egg~activity_scores, data = A.P.8)
Anova(linearmodel, type = 3)
linearmodel<-lm(total_egg~activity_scores, data = A.P.10)
Anova(linearmodel, type = 3)
linearmodel<-lm(total_egg~activity_scores, data = A.P.11)
Anova(linearmodel, type = 3)
linearmodel<-lm(total_egg~activity_scores, data = A.P.12)
Anova(linearmodel, type = 3)
```

Exploration:

```
linearmodel<-lm(total_egg~exploration_scores, data = A.P.2)
Anova(linearmodel, type = 3)
linearmodel<-lm(total_egg~exploration_scores, data = A.P.3)
Anova(linearmodel, type = 3)
linearmodel<-lm(total_egg~exploration_scores, data = A.P.5)
Anova(linearmodel, type = 3)
linearmodel<-lm(total_egg~exploration_scores, data = A.P.8)
Anova(linearmodel, type = 3)
linearmodel<-lm(total_egg~exploration_scores, data = A.P.10)
Anova(linearmodel, type = 3)
linearmodel<-lm(total_egg~exploration_scores, data = A.P.11)
Anova(linearmodel, type = 3)
linearmodel<-lm(total_egg~exploration_scores, data = A.P.12)
Anova(linearmodel, type = 3)
```

Part 5: Personality types and prey stage preference (Manly index)

Pearson correlations:

Activity:

```
linearmodel<-lm(Manly_index_1~activity_scores, data = A.P)
Anova(linearmodel, type = 3)
linearmodel<-lm(Manly_index_1~activity_scores, data = A.P.2)
Anova(linearmodel, type = 3)
linearmodel<-lm(Manly_index_1~activity_scores, data = A.P.3)
Anova(linearmodel, type = 3)
linearmodel<-lm(Manly_index_1~activity_scores, data = A.P.4)
```

```
Anova(linearmodel, type = 3)
linearmodel<-lm(Manly_index_1~activity_scores, data = A.P.5)
Anova(linearmodel, type = 3)
linearmodel<-lm(Manly_index_1~activity_scores, data = A.P.6)
Anova(linearmodel, type = 3)
linearmodel<-lm(Manly_index_1~activity_scores, data = A.P.7)
Anova(linearmodel, type = 3)
linearmodel<-lm(Manly_index_1~activity_scores, data = A.P.8)
Anova(linearmodel, type = 3)
linearmodel<-lm(Manly_index_1~activity_scores, data = A.P.9)
Anova(linearmodel, type = 3)
linearmodel<-lm(Manly_index_1~activity_scores, data = A.P.10)
Anova(linearmodel, type = 3)
linearmodel<-lm(Manly_index_1~activity_scores, data = A.P.11)
Anova(linearmodel, type = 3)
linearmodel<-lm(Manly_index_1~activity_scores, data = A.P.12)
Anova(linearmodel, type = 3)
linearmodel<-lm(Manly_index_1~activity_scores, data = A.P.13)
Anova(linearmodel, type = 3)
linearmodel<-lm(Manly_index_1~activity_scores, data = A.P.14)
Anova(linearmodel, type = 3)
linearmodel<-lm(Manly_index_1~activity_scores, data = A.P.15)
Anova(linearmodel, type = 3)
```

Exploration:

```
linearmodel<-lm(Manly_index_1~exploration_scores, data = A.P)
Anova(linearmodel, type = 3)
linearmodel<-lm(Manly_index_1~exploration_scores, data = A.P.2)
Anova(linearmodel, type = 3)
linearmodel<-lm(Manly_index_1~exploration_scores, data = A.P.3)
Anova(linearmodel, type = 3)
linearmodel<-lm(Manly_index_1~exploration_scores, data = A.P.4)
Anova(linearmodel, type = 3)
linearmodel<-lm(Manly_index_1~exploration_scores, data = A.P.5)
Anova(linearmodel, type = 3)
linearmodel<-lm(Manly_index_1~exploration_scores, data = A.P.6)
Anova(linearmodel, type = 3)
linearmodel<-lm(Manly_index_1~exploration_scores, data = A.P.7)
Anova(linearmodel, type = 3)
linearmodel<-lm(Manly_index_1~exploration_scores, data = A.P.8)
Anova(linearmodel, type = 3)
linearmodel<-lm(Manly_index_1~exploration_scores, data = A.P.9)
Anova(linearmodel, type = 3)
linearmodel<-lm(Manly_index_1~exploration_scores, data = A.P.10)
Anova(linearmodel, type = 3)
linearmodel<-lm(Manly_index_1~exploration_scores, data = A.P.11)
Anova(linearmodel, type = 3)
linearmodel<-lm(Manly_index_1~exploration_scores, data = A.P.12)
Anova(linearmodel, type = 3)
linearmodel<-lm(Manly_index_1~exploration_scores, data = A.P.13)
Anova(linearmodel, type = 3)
```

```
linearmodel<-lm(Manly_index_1~exploration_scores, data = A.P.14)
Anova(linearmodel, type = 3)
linearmodel<-lm(Manly_index_1~exploration_scores, data = A.P.15)
Anova(linearmodel, type = 3)
```

Quadratic regression:

Activity:

```
quadraticmodel<-lm(Manly_index_1~activity_scores+activity_scores2, data =
A.P)
Anova(quadraticmodel, type = 3)
quadraticmodel<-lm(Manly_index_1~activity_scores+activity_scores2, data =
A.P.2)
Anova(quadraticmodel, type = 3)
quadraticmodel<-lm(Manly_index_1~activity_scores+activity_scores2, data =
A.P.3)
Anova(quadraticmodel, type = 3)
quadraticmodel<-lm(Manly_index_1~activity_scores+activity_scores2, data =
A.P.4)
Anova(quadraticmodel, type = 3)
quadraticmodel<-lm(Manly_index_1~activity_scores+activity_scores2, data =
A.P.5)
Anova(quadraticmodel, type = 3)
quadraticmodel<-lm(Manly_index_1~activity_scores+activity_scores2, data =
A.P.6)
Anova(quadraticmodel, type = 3)
quadraticmodel<-lm(Manly_index_1~activity_scores+activity_scores2, data =
A.P.7)
Anova(quadraticmodel, type = 3)
quadraticmodel<-lm(Manly_index_1~activity_scores+activity_scores2, data =
A.P.8)
Anova(quadraticmodel, type = 3)
quadraticmodel<-lm(Manly_index_1~activity_scores+activity_scores2, data =
A.P.9)
Anova(quadraticmodel, type = 3)
quadraticmodel<-lm(Manly_index_1~activity_scores+activity_scores2, data =
A.P.10)
Anova(quadraticmodel, type = 3)
quadraticmodel<-lm(Manly_index_1~activity_scores+activity_scores2, data =
A.P.11)
Anova(quadraticmodel, type = 3)
quadraticmodel<-lm(Manly_index_1~activity_scores+activity_scores2, data =
A.P.12)
Anova(quadraticmodel, type = 3)
quadraticmodel<-lm(Manly_index_1~activity_scores+activity_scores2, data =
A.P.13)
Anova(quadraticmodel, type = 3)
quadraticmodel<-lm(Manly_index_1~activity_scores+activity_scores2, data =
A.P.14)
Anova(quadraticmodel, type = 3)
quadraticmodel<-lm(Manly_index_1~activity_scores+activity_scores2, data =
```

```
A.P.15)
Anova(quadraticmodel, type = 3)
```

Exploration:

```
quadraticmodel.1<-lm(Manly_index_1~exploration_scores+exploration_scores2,
data = A.P)
Anova(quadraticmodel.1, type = 3)
quadraticmodel.1<-lm(Manly_index_1~exploration_scores+exploration_scores2,
data = A.P.2)
Anova(quadraticmodel.1, type = 3)
quadraticmodel.1<-lm(Manly_index_1~exploration_scores+exploration_scores2,
data = A.P.5)
Anova(quadraticmodel.1, type = 3)
quadraticmodel.1<-lm(Manly_index_1~exploration_scores+exploration_scores2,
data = A.P.8)
Anova(quadraticmodel.1, type = 3)
quadraticmodel.1<-lm(Manly_index_1~exploration_scores+exploration_scores2,
data = A.P.10)
Anova(quadraticmodel.1, type = 3)
quadraticmodel.1<-lm(Manly_index_1~exploration_scores+exploration_scores2,
data = A.P.11)
Anova(quadraticmodel.1, type = 3)
quadraticmodel.1<-lm(Manly_index_1~exploration_scores+exploration_scores2,
data = A.P.12)
Anova(quadraticmodel.1, type = 3)
quadraticmodel.1<-lm(Manly_index_1~exploration_scores+exploration_scores2,
data = A.P.14)
Anova(quadraticmodel.1, type = 3)
quadraticmodel.1<-lm(Manly_index_1~exploration_scores+exploration_scores2,
data = A.P.15)
Anova(quadraticmodel.1, type = 3)
```

PP+PM.E

2024-12-05

Early life effects

Dataset

```
A.E <- read.csv("Activity.E.old.csv", header=TRUE)
A.E.2 <- subset(A.E, sex%in% "female")
A.E.3 <- subset(A.E, species%in% "persimilis" & sex%in% "female"&
treatment%in% "egg")
A.E.4 <- subset(A.E, species%in% "persimilis" & sex%in% "male"& treatment%in%
"egg")
A.E.5 <- subset(A.E, species%in% "macropilis" & sex%in% "female"&
treatment%in% "egg")
A.E.6 <- subset(A.E, species%in% "macropilis" & sex%in% "male"& treatment%in%
"egg")
A.E.7 <- subset(A.E, species%in% "macropilis"& sex%in% "male"& treatment%in%
"mobile")
A.E.8 <- subset(A.E, species%in% "persimilis" & sex%in% "female"&
treatment%in% "mobile")
A.E.9 <- subset(A.E, species%in% "persimilis" & sex%in% "male"& treatment%in%
"mobile")
A.E.10 <- subset(A.E, species%in% "macropilis" & sex%in% "female"&
treatment%in% "mobile")
A.E.11 <- subset(A.E, species%in% "macropilis")
A.E.12 <- subset(A.E, species%in% "persimilis")
A.E.13 <- subset(A.E, sex%in% "male")
A.E.14 <- subset(A.E, treatment%in% "egg")
A.E.15 <- subset(A.E, treatment%in% "mobile")

A.E$activity_scores2<-A.E$activity_scores^2
A.E.2$activity_scores2<-A.E.2$activity_scores^2
A.E.3$activity_scores2<-A.E.3$activity_scores^2
A.E.4$activity_scores2<-A.E.4$activity_scores^2
A.E.5$activity_scores2<-A.E.5$activity_scores^2
A.E.6$activity_scores2<-A.E.6$activity_scores^2
A.E.7$activity_scores2<-A.E.7$activity_scores^2
A.E.8$activity_scores2<-A.E.8$activity_scores^2
A.E.9$activity_scores2<-A.E.9$activity_scores^2
A.E.10$activity_scores2<-A.E.10$activity_scores^2
A.E.11$activity_scores2<-A.E.11$activity_scores^2
A.E.12$activity_scores2<-A.E.12$activity_scores^2
A.E.13$activity_scores2<-A.E.13$activity_scores^2
A.E.14$activity_scores2<-A.E.14$activity_scores^2
A.E.15$activity_scores2<-A.E.15$activity_scores^2

A.E$exploration_scores2<-A.E$exploration_scores^2
A.E.2$exploration_scores2<-A.E.2$exploration_scores^2
```

```
A.E.3$exploration_scores2<-A.E.3$exploration_scores^2
A.E.4$exploration_scores2<-A.E.4$exploration_scores^2
A.E.5$exploration_scores2<-A.E.5$exploration_scores^2
A.E.6$exploration_scores2<-A.E.6$exploration_scores^2
A.E.7$exploration_scores2<-A.E.7$exploration_scores^2
A.E.8$exploration_scores2<-A.E.8$exploration_scores^2
A.E.9$exploration_scores2<-A.E.9$exploration_scores^2
A.E.10$exploration_scores2<-A.E.10$exploration_scores^2
A.E.11$exploration_scores2<-A.E.11$exploration_scores^2
A.E.12$exploration_scores2<-A.E.12$exploration_scores^2
A.E.13$exploration_scores2<-A.E.13$exploration_scores^2
A.E.14$exploration_scores2<-A.E.14$exploration_scores^2
A.E.15$exploration_scores2<-A.E.15$exploration_scores^2
```

Part 1: Population mean

Manly's index in the first assay:

```
manly.1.end<-glm(Manly_index_1~(treatment*species*sex)-
treatment:species:sex,data = A.E, family =gaussian(link=identity))
Anova(manly.1.end, type=3)
```

Remove non significant interactions:

```
manly.1.end.1<-glm(Manly_index_1~treatment+species+sex,data = A.E, family
=gaussian(link=identity))
Anova(manly.1.end.1, type=3)
```

Mean activity in the first assay:

```
activity.1<-glm(proportion_moving_1~treatment*species*sex-
treatment:species:sex, data=A.E, family = quasibinomial)
Anova(activity.1,type=3)
```

Remove non significant interactions:

```
activity.2<-glm(proportion_moving_1~treatment+species+sex, data=A.E, family =
quasibinomial)
Anova(activity.2,type=3)
```

Mean exploration in the first assay:

```
exploration.2<-glm(no_changes~treatment*species*sex-treatment:species:sex,
data=A.E, family = poisson(link = "log"))
Anova(exploration.2,type=3)
```

Remove non significant interactions:

```
exploration.3<-glm(no_changes~treatment+species+sex, data=A.E, family =
poisson(link = "log"))
Anova(exploration.3,type=3)
```

Total prey consumption in the first two assays:

```
m5.2<- glm(prey_eaten ~ treatment*species*sex-treatment:species:sex,
data=A.E, family=gaussian(link=identity))
Anova(m5.2, type=3)
```

Remove non significant interactions:

```
m5.3<- glm(prey_eaten ~ treatment+species+sex+species:sex, data=A.E,
family=gaussian(link=identity))
Anova(m5.3, type=3)
```

Total egg laying:

```
egg.laying <- glm(total_egg ~ treatment*species, data=A.E.2,family =
gaussian(link=identity))
Anova(egg.laying, type=3)
```

Remove non significant interactions:

```
egg.laying <- glm(total_egg ~ treatment+species, data=A.E.2,family =
gaussian(link=identity))
Anova(egg.laying, type = 3)
```

Part 2: Personality expression (Intraclass correlation coefficients - ICCs)

Personality in activity:

```
R.1<-icc(cbind(A.E$proportion_moving_1,A.E$proportion_moving_2),model =
"twoway", unit = "average")
print(R.1)
R.2<-icc(cbind(A.E.2$proportion_moving_1,A.E.2$proportion_moving_2),model =
"twoway", unit = "average")
print(R.2)
R.3<-icc(cbind(A.E.3$proportion_moving_1,A.E.3$proportion_moving_2),model =
"twoway", unit = "average")
print(R.3)
R.4<-icc(cbind(A.E.4$proportion_moving_1,A.E.4$proportion_moving_2),model =
"twoway", unit = "average")
print(R.4)
R.5<-icc(cbind(A.E.5$proportion_moving_1,A.E.5$proportion_moving_2),model =
"twoway", unit = "average")
print(R.5)
R.6<-icc(cbind(A.E.6$proportion_moving_1,A.E.6$proportion_moving_2),model =
"twoway", unit = "average")
print(R.6)
R.7<-icc(cbind(A.E.7$proportion_moving_1,A.E.7$proportion_moving_2),model =
"twoway", unit = "average")
print(R.7)
R.8<-icc(cbind(A.E.8$proportion_moving_1,A.E.8$proportion_moving_2),model =
"twoway", unit = "average")
```

```
print(R.8)
R.9<-icc(cbind(A.E.9$proportion_moving_1,A.E.9$proportion_moving_2),model =
"twoway", unit = "average")
print(R.9)
R.10<-icc(cbind(A.E.10$proportion_moving_1,A.E.10$proportion_moving_2),model
= "twoway", unit = "average")
print(R.10)
R.11<-icc(cbind(A.E.11$proportion_moving_1,A.E.11$proportion_moving_2),model
= "twoway", unit = "average")
print(R.11)
R.12<-icc(cbind(A.E.12$proportion_moving_1,A.E.12$proportion_moving_2),model
= "twoway", unit = "average")
print(R.12)
R.13<-icc(cbind(A.E.13$proportion_moving_1,A.E.13$proportion_moving_2),model
= "twoway", unit = "average")
print(R.13)
R.14<-icc(cbind(A.E.14$proportion_moving_1,A.E.14$proportion_moving_2),model
= "twoway", unit = "average")
print(R.14)
R.15<-icc(cbind(A.E.15$proportion_moving_1,A.E.15$proportion_moving_2),model
= "twoway", unit = "average")
print(R.15)
```

Personality in exploration:

```
R.1<-icc(cbind(A.E$no_changes,sqrt(A.E$no_places_3)),model = "twoway", unit =
"average")
print(R.1)
R.2<-icc(cbind(A.E.2$no_changes,sqrt(A.E.2$no_places_3)),model = "twoway",
unit = "average")
print(R.2)
R.3<-icc(cbind(A.E.3$no_changes,sqrt(A.E.3$no_places_3)),model = "twoway",
unit = "average")
print(R.3)
R.4<-icc(cbind(A.E.4$no_changes,sqrt(A.E.4$no_places_3)),model = "twoway",
unit = "average")
print(R.4)
R.5<-icc(cbind(A.E.5$no_changes,sqrt(A.E.5$no_places_3)),model = "twoway",
unit = "average")
print(R.5)
R.6<-icc(cbind(A.E.6$no_changes,sqrt(A.E.6$no_places_3)),model = "twoway",
unit = "average")
print(R.6)
R.7<-icc(cbind(A.E.7$no_changes,sqrt(A.E.7$no_places_3)),model = "twoway",
unit = "average")
print(R.7)
R.8<-icc(cbind(A.E.8$no_changes,sqrt(A.E.8$no_places_3)),model = "twoway",
unit = "average")
print(R.8)
R.9<-icc(cbind(A.E.9$no_changes,sqrt(A.E.9$no_places_3)),model = "twoway",
unit = "average")
```

```
print(R.9)
R.10<-icc(cbind(A.E.10$no_changes,sqrt(A.E.10$no_places_3)),model = "twoway",
unit = "average")
print(R.10)
R.11<-icc(cbind(A.E.11$no_changes,sqrt(A.E.11$no_places_3)),model = "twoway",
unit = "average")
print(R.11)
R.12<-icc(cbind(A.E.12$no_changes,sqrt(A.E.12$no_places_3)),model = "twoway",
unit = "average")
print(R.12)
R.13<-icc(cbind(A.E.13$no_changes,sqrt(A.E.13$no_places_3)),model = "twoway",
unit = "average")
print(R.13)
R.14<-icc(cbind(A.E.14$no_changes,sqrt(A.E.14$no_places_3)),model = "twoway",
unit = "average")
print(R.14)
R.15<-icc(cbind(A.E.15$no_changes,sqrt(A.E.15$no_places_3)),model = "twoway",
unit = "average")
print(R.15)
```

Part 3: Personality composition

Personality composition in activity:

```
activity.1.1<-glm(activity_scores~treatment*species*sex,data = A.E, family
=poisson(link=log))
Anova(activity.1.1,type=3)
```

Remove non significant interactions:

```
activity.2.1<-glm(activity_scores~treatment+species+sex,data = A.E, family
=poisson(link=log))
Anova(activity.2.1,type=3)
```

Personality composition in exploration:

```
ex.1.2<-glm(exploration_scores~treatment*species*sex,data = A.E, family
=poisson(link=log))
Anova(ex.1.2,type=3 )
```

Remove non significant interactions:

```
ex.2.2<-glm(exploration_scores~treatment+species+sex+species:sex,data = A.E,
family =poisson(link=log))
Anova(ex.2.2,type=3 )
```

Part 4: Personality types and short-term fitness

Activity:

```
linearmodel<-lm(total_egg~activity_scores, data = A.E.2)
Anova(linearmodel, type = 3)
linearmodel<-lm(total_egg~activity_scores, data = A.E.3)
Anova(linearmodel, type = 3)
linearmodel<-lm(total_egg~activity_scores, data = A.E.5)
Anova(linearmodel, type = 3)
linearmodel<-lm(total_egg~activity_scores, data = A.E.8)
Anova(linearmodel, type = 3)
linearmodel<-lm(total_egg~activity_scores, data = A.E.10)
Anova(linearmodel, type = 3)
linearmodel<-lm(total_egg~activity_scores, data = A.E.11)
Anova(linearmodel, type = 3)
linearmodel<-lm(total_egg~activity_scores, data = A.E.12)
Anova(linearmodel, type = 3)
```

Exploration:

```
linearmodel<-lm(total_egg~exploration_scores, data = A.E.2)
Anova(linearmodel, type = 3)
linearmodel<-lm(total_egg~exploration_scores, data = A.E.3)
Anova(linearmodel, type = 3)
linearmodel<-lm(total_egg~exploration_scores, data = A.E.5)
Anova(linearmodel, type = 3)
linearmodel<-lm(total_egg~exploration_scores, data = A.E.8)
Anova(linearmodel, type = 3)
linearmodel<-lm(total_egg~exploration_scores, data = A.E.10)
Anova(linearmodel, type = 3)
linearmodel<-lm(total_egg~exploration_scores, data = A.E.11)
Anova(linearmodel, type = 3)
linearmodel<-lm(total_egg~exploration_scores, data = A.E.12)
Anova(linearmodel, type = 3)
```

Part 5: Personality types and prey stage preference (Manly index)

Pearson correlations:

Activity:

```
linearmodel<-lm(Manly_index_1~activity_scores, data = A.E)
Anova(linearmodel, type = 3)
linearmodel<-lm(Manly_index_1~activity_scores, data = A.E.2)
Anova(linearmodel, type = 3)
linearmodel<-lm(Manly_index_1~activity_scores, data = A.E.3)
Anova(linearmodel, type = 3)
linearmodel<-lm(Manly_index_1~activity_scores, data = A.E.4)
```

```
Anova(linearmodel, type = 3)
linearmodel<-lm(Manly_index_1~activity_scores, data = A.E.5)
Anova(linearmodel, type = 3)
linearmodel<-lm(Manly_index_1~activity_scores, data = A.E.6)
Anova(linearmodel, type = 3)
linearmodel<-lm(Manly_index_1~activity_scores, data = A.E.7)
Anova(linearmodel, type = 3)
linearmodel<-lm(Manly_index_1~activity_scores, data = A.E.8)
Anova(linearmodel, type = 3)
linearmodel<-lm(Manly_index_1~activity_scores, data = A.E.9)
Anova(linearmodel, type = 3)
linearmodel<-lm(Manly_index_1~activity_scores, data = A.E.10)
Anova(linearmodel, type = 3)
linearmodel<-lm(Manly_index_1~activity_scores, data = A.E.11)
Anova(linearmodel, type = 3)
linearmodel<-lm(Manly_index_1~activity_scores, data = A.E.12)
Anova(linearmodel, type = 3)
linearmodel<-lm(Manly_index_1~activity_scores, data = A.E.13)
Anova(linearmodel, type = 3)
linearmodel<-lm(Manly_index_1~activity_scores, data = A.E.14)
Anova(linearmodel, type = 3)
linearmodel<-lm(Manly_index_1~activity_scores, data = A.E.15)
Anova(linearmodel, type = 3)
```

Exploration:

```
linearmodel<-lm(Manly_index_1~exploration_scores, data = A.E)
Anova(linearmodel, type = 3)
linearmodel<-lm(Manly_index_1~exploration_scores, data = A.E.2)
Anova(linearmodel, type = 3)
linearmodel<-lm(Manly_index_1~exploration_scores, data = A.E.3)
Anova(linearmodel, type = 3)
linearmodel<-lm(Manly_index_1~exploration_scores, data = A.E.4)
Anova(linearmodel, type = 3)
linearmodel<-lm(Manly_index_1~exploration_scores, data = A.E.5)
Anova(linearmodel, type = 3)
linearmodel<-lm(Manly_index_1~exploration_scores, data = A.E.7)
Anova(linearmodel, type = 3)
linearmodel<-lm(Manly_index_1~exploration_scores, data = A.E.8)
Anova(linearmodel, type = 3)
linearmodel<-lm(Manly_index_1~exploration_scores, data = A.E.9)
Anova(linearmodel, type = 3)
linearmodel<-lm(Manly_index_1~exploration_scores, data = A.E.10)
Anova(linearmodel, type = 3)
linearmodel<-lm(Manly_index_1~exploration_scores, data = A.E.11)
Anova(linearmodel, type = 3)
linearmodel<-lm(Manly_index_1~exploration_scores, data = A.E.12)
Anova(linearmodel, type = 3)
linearmodel<-lm(Manly_index_1~exploration_scores, data = A.E.13)
Anova(linearmodel, type = 3)
linearmodel<-lm(Manly_index_1~exploration_scores, data = A.E.14)
Anova(linearmodel, type = 3)
```

```
linearmodel<-lm(Manly_index_1~exploration_scores, data = A.E.15)
Anova(linearmodel, type = 3)
```

Quadratic regression:

Activity:

```
quadraticmodel<-lm(Manly_index_1~activity_scores+activity_scores2, data =
A.E)
Anova(quadraticmodel, type = 3)
quadraticmodel<-lm(Manly_index_1~activity_scores+activity_scores2, data =
A.E.2)
Anova(quadraticmodel, type = 3)
quadraticmodel<-lm(Manly_index_1~activity_scores+activity_scores2, data =
A.E.3)
Anova(quadraticmodel, type = 3)
quadraticmodel<-lm(Manly_index_1~activity_scores+activity_scores2, data =
A.E.4)
Anova(quadraticmodel, type = 3)
quadraticmodel<-lm(Manly_index_1~activity_scores+activity_scores2, data =
A.E.5)
Anova(quadraticmodel, type = 3)
quadraticmodel<-lm(Manly_index_1~activity_scores+activity_scores2, data =
A.E.6)
Anova(quadraticmodel, type = 3)
quadraticmodel<-lm(Manly_index_1~activity_scores+activity_scores2, data =
A.E.7)
Anova(quadraticmodel, type = 3)
quadraticmodel<-lm(Manly_index_1~activity_scores+activity_scores2, data =
A.E.8)
Anova(quadraticmodel, type = 3)
quadraticmodel<-lm(Manly_index_1~activity_scores+activity_scores2, data =
A.E.9)
Anova(quadraticmodel, type = 3)
quadraticmodel<-lm(Manly_index_1~activity_scores+activity_scores2, data =
A.E.10)
Anova(quadraticmodel, type = 3)
quadraticmodel<-lm(Manly_index_1~activity_scores+activity_scores2, data =
A.E.11)
Anova(quadraticmodel, type = 3)
quadraticmodel<-lm(Manly_index_1~activity_scores+activity_scores2, data =
A.E.12)
Anova(quadraticmodel, type = 3)
quadraticmodel<-lm(Manly_index_1~activity_scores+activity_scores2, data =
A.E.13)
Anova(quadraticmodel, type = 3)
quadraticmodel<-lm(Manly_index_1~activity_scores+activity_scores2, data =
A.E.14)
Anova(quadraticmodel, type = 3)
quadraticmodel<-lm(Manly_index_1~activity_scores+activity_scores2, data =
A.E.15)
Anova(quadraticmodel, type = 3)
```

Exploration:

```
quadraticmodel.1<-lm(Manly_index_1~exploration_scores+exploration_scores2,
data = A.E)
Anova(quadraticmodel.1, type = 3)
quadraticmodel.1<-lm(Manly_index_1~exploration_scores+exploration_scores2,
data = A.E.2)
Anova(quadraticmodel.1, type = 3)
quadraticmodel.1<-lm(Manly_index_1~exploration_scores+exploration_scores2,
data = A.E.5)
Anova(quadraticmodel.1, type = 3)
quadraticmodel.1<-lm(Manly_index_1~exploration_scores+exploration_scores2,
data = A.E.10)
Anova(quadraticmodel.1, type = 3)
quadraticmodel.1<-lm(Manly_index_1~exploration_scores+exploration_scores2,
data = A.E.11)
Anova(quadraticmodel.1, type = 3)
quadraticmodel.1<-lm(Manly_index_1~exploration_scores+exploration_scores2,
data = A.E.14)
Anova(quadraticmodel.1, type = 3)
quadraticmodel.1<-lm(Manly_index_1~exploration_scores+exploration_scores2,
data = A.E.15)
Anova(quadraticmodel.1, type = 3)
```
